# Profiling leadership: Attitudes, knowledge and training in the biological sciences

**DOI:** 10.1371/journal.pone.0286826

**Published:** 2023-06-07

**Authors:** James A. L. Brown

**Affiliations:** 1 Department of Biological Science, University of Limerick, Limerick, Ireland; 2 Limerick Digital Cancer Research Centre (LDCRC), Health Research Institute (HRI), Bernal Institute, University of Limerick, Limerick, Ireland; COMSATS University Islamabad - Wah Campus, PAKISTAN

## Abstract

The development and practice of good leadership skills (distinct from management skills) enhances both an individual’s career development, and their organization. However, universities are known to present unique issues around the development, and practice, of good leadership. Good leadership skills should be considered essential for university staff who train (and mentor) staff or students. Currently, there is no clear evidence that staff in the biological (life) sciences undergo formal (routine) leadership skills training (or appraisal). Furthermore, what leadership training this group needs, or wants, is unknown. A questionnaire was designed to explore leadership dimensions (roles, training, perceptions, and attitudes), and incorporated the Leadership Attitudes and Belief scale (LABS) instrument. Including LABS allows evaluation of leadership attitudes as either Systemic (individual responsibility) or Hierarchical (chain-of-command). Self-selecting biological science academics and staff were recruited using an online survey. Analysis focused on academic staff (lecturer/Assistant professor, and above), and explored the relationship of leadership dimensions with key categories (career stage, gender, age, role, and professional experience). Staff were found to be knowledgeable about what leadership is, but strongly desire formal training in leadership skills and practice. Importantly, staff did not have access to specific leadership training (but did have access to management training), but felt strongly that gaining leadership skills would improve their professional skill set. Analysis found that academics in the biological sciences were oriented towards Systemic leadership, a more collective and supportive approach. It was clear that while good leadership skills are highly valued by academic staff, in practice these skills are underprovided in the biological sciences workplace. This work provides a profile, and benchmark, of leadership (current skills, and desired needs) in the biological sciences. These results provide evidence for the need to embed specific leadership skills training into professional development (and teaching) programmes in the biological sciences.

## Introduction

Universities (including higher/third level educational and research institutions) are large hierarchical organizations that encompass management and leadership at every level from the macro (leading colleagues, faculties, departments) down to the micro (classes, research groups, small groups, or individual undergraduate students) [1–3]. It has been shown that generally, people with a positive opinion toward a leader often have aligned attitudinal factors (including cognition and behaviors) [4]. Investigating and defining these leadership attitudes in specific contexts (including departments, or roles/positions) within the university is beneficial for improving an individuals’ personal and professional development, and for improving the efficacy and atmosphere within the whole organization.

The influence of effective leadership in (scientific) research groups, and the host organization, is recognized as a key element of a successful research group [5–7]. However, it has been reported that current senior academic staff (e.g. full professors) are unsure what “leadership” is, and were poorly prepared for leadership roles [8]. Increasingly, publication metrics are being used as a “measure” of leadership, underscoring how poorly the concept of leadership is understood [9]. This is supported by the relaxed conceptual division between the definitions of management (focused on organizational tasks, results, methods and goals) and leadership (focusing on personal relationships and organizing individuals) [3], which results in a substitution of management/administrative skills training for leadership skill training at a functional level.

While definitions of leadership can be variable [10], here we use the definition of Leadership as: “an emergent process characterized by the tension between systemic (relational) and hierarchical (positional leader) processes” (from [11]). Furthermore, we use the definition of Leadership Development as: “a long-term process incorporating personal experience, interpersonal skills, and self-analysis, focused on enhancing leadership capacity” [11, 12]. The Leadership Attitudes and Belief scale (LABS) is a useful system to understand and categorize leadership, and places an individuals leadership attitudes and beliefs into either a Systemic or Hierarchical group [11, 13, 14]. Hierarchical leadership groups believe organizational leadership follows a chain-of-command (position based), and employs the traditional pyramid-shaped top-down leadership structure. Systemic leadership groups believe individuals take responsibility for organizational leadership, and considers the whole organizational context/system (past, present, future) and how developing relationships between people allows large, complex problems to be solved (which was not possible individually). Importantly, we can define a leader’s role in their organization as effectively optimizing and combining the best of these two approaches [10].

Currently, of the STEMM subjects (Science, Technology, Engineering, Mathematics, Medicine) there is no clear evidence that staff in the biological (life) Sciences undergo specific leadership skills training or development (including evaluation), or what leadership training this group needs or desires. Quantifying this knowledge gap in the leadership training and skills development needs of the biological sciences will bring the biological sciences in line with existing work in other STEMM subjects in developing, deploying and evaluating leadership frameworks and skills [15–18]). Focusing on the biological sciences (concentrating on academic staff), this study examines and evaluates (quantitatively and qualitatively) individuals’ leadership roles, knowledge, skills, perceptions and attitudes within the university sector. Additionally, the desire for leadership training is explored. Profiling the current leadership landscape in the biological sciences will inform the creation of a detailed and targeted leadership knowledge and skills program, specific and relevant to the needs and requirements of academics within the biological sciences.

## Material and methods

### Questionnaire design

Prior to beginning research-focused questions, participants must actively accept the informed consent, and ethics statements (see Ethical Statement section). The questionnaire (S1 Appendix) was designed with questions grouped by section, exploring five key areas of leadership: Demographics (quantitative), “What do I think leadership is?” (qualitative), Skills/training/knowledge (quantitative and qualitative), Attitudes (quantitative and qualitative), Perceptions (quantitative and qualitative). Attitudes and Perceptions sections each incorporated questions modified from The Leadership Attitudes and Beliefs Scale III (LABS) questionnaire [14, 19]. Permission to use the LABS questionnaire was granted by Professor Richard Wielkiewicz (The College of Saint Benedict and Saint John’s University, USA; personal correspondence). Response options collected include answers categorised using a 5-point Likert Scale (“strongly agree” to “strongly disagree”), binary answers (yes/no), qualitative responses (free text), and categorical data (including professional role, age group, geographic location).

### Participants and recruitment

The questionnaire was administered using a fully anonymous online survey/questionnaire (using surveymonkey.com). Participants were recruited in the first quarter of 2021 using publicly available worldwide posts (containing the survey link) to social media/networking platforms (previously shown to be effective [20]), including Twitter, LinkedIn, and restricted access scientific networks (including Slack and Discord platforms) from the author’s accounts on each platform. It is acknowledged that this recruitment method means the cohort/sample/population were self-selecting, non-random, fully voluntarily, and explicitly within the biological sciences (as indicated by given occupation/position question answers).

To amplify the international reach of the survey posts on twitter, posts utilized mentions of relevant hashtags (including: #research #ECR #AcademicChatter #AcademicTwitter #faculty #Science #cellBiology #Biochemistry #lecturer #educator), and tagged community-specific twitter accounts (including: @academicChatter @OpenAcademics @embl @timeshighered @hapyresearchers @PPPublishing @Science @EACRnews @FNucleosome @HHMINEWS).

### Data and statistical analysis

Answering individual questions was not mandatory/enforced, the number of responses to individual questions varied, with percentages and numbers of responses (n) to each question indicated. Likert Scale Data was converted using the sliding scale: 5 = strongly agree/very likely, down to 1 = strongly disagree/very unlikely. Quantitative analysis of free-text responses utilized word-cloud frequencies of aggregated free-text responses. Qualitative analysis included participants own free-text responses, paraphrased free-text summaries of key or common responses, and word-clouds. Data was analyzed using GraphPad Prism (Spearman’s non-parametric correlation, two-tailed test; and One-way ANOVA Kruskal-Wallis test with a Dunns post-test) with p>0.05 significant, and p>0.005 highly significant.

### Ethical statement

Project Ethical approval was granted by the University of Limerick Research Ethics Committee (2021_03_06_S&E). The online questionnaire’s cover page provided participant study information, including study “eligibility”, study “purpose”, study “procedure and withdrawal” and study contact statements. Participants were informed that involvement was voluntary, confirmed they were over 18 years old, and agreed to participation by selecting an affirmative checkbox. A second Ethical consent (page) provided more specific ethics statements, including informed consent, publication, data anonymization statements, and withdrawal information. Written informed consent was provided by filling in a checkbox to indicate that participants had read and accepted the form and agreed to participate in the study (complete forms and questionnaire see **S1 Appendix**).

## Results

### Cohort description

To contextualize the project, worldwide recruitment (to the online questionnaire) was performed during the COVID pandemic (worldwide many countries were in some form of lockdown- often a second or third, and worldwide many universities/research institutions were primarily/exclusively working from home for >12 months). Importantly, as enforced/mandatory answering of every survey question was not compulsory, response rates for individual questions varied (and are indicated).

Participants predominantly identified as female 88% (n = 15), with 12% (n = 2) male. No participants selected any other gender options provided (S1 Table). Exploring the age range of respondents >44% (n = 19) were >40 years old, followed by >23% (n = 10) in the 35–40 year-old bracket. ~7% (n = 3) were 18–24 years old (S2 Table). >65% of respondents were 35 years or older, consistent with the career stage profile, and consistent with previous reviews of the sector [21]. Geographically, the majority of participants were from western Europe ~ 60%, followed by Oceania ~16% and North America ~12% (S3 Table). There were n = 42 responses self-identifying their job title/role, ~36% Academics (n = 15), ~26% Postdoctoral researchers (n = 11) and ~19% Research-focused Group leaders (n = 8) (S4 Table). Postgraduate students (masters and PhD) accounted for ~14% (n = 6), and undergraduate students <5% (n = 2). There was ~2% (n = 1) respondents’ who identified as professional staff. Early career researchers (ECR, 0–10 years post PhD) made up >55% of the respondent pool, spread between 6–10 years (~31%) and 0–5 years (25%) post PhD. Mid-career researchers (11–15 years post PhD) made up ~14% of respondents, and >27% of participants would be considered senior (>16 years post PhD) (S5 Table) [20].

Exploring participants’ role/position (n = 40), 52.5% (n = 21) of respondents identified with academic titles (Lecturer, Senior lecturer, Professor), which rises to 57.5% (n = 23) including the “other” answer category of PI (Principal Investigator, e.g., leader of a research group) (n = 2). Including non-faculty academic staff (postdoctoral researchers) 85% (n = 34) could be considered academic (Table 1). Interestingly, 15% (n = 6) selected the other response option, composed of Staff scientist (n = 1), Pharma industry (n = 1), postgraduate research students (n = 2), and unidentified (n = 2).

**Table 1:** Participant job title.

Response	%	n =
Professor	17.5%	7
Assistant Professor	5.0%	2
Senior Lecturer	10%	4
Lecturer	20%	8
Postdoctoral Researcher	27.5%	11
Other (please specify)	15%	6
Other (PI)_	5.0%	2
	**Total**	**40**

### Existing leadership positions/responsibilities

Exploring leadership positions/responsibilities of the cohort, >51% (n = 22) identified as engaged in the management of a research group (S6 Table). Exploring the participants responsibilities ~78% (n = 33) self-identified as members of management groups (committees, professional councils/boards, etc.), with ~40% (n = 17) indicating they were not (Table 2). Of these the majority 58% (n = 25) sat on management groups internal to their organization. Furthermore >69% (n = 30) of those leading research groups indicated they were supervising undergraduate students (e.g., final year projects). Those supervised included >23% postdoctoral researchers (n = 22), and ~50% postgraduate students (~30%, n = 28, PhD students; >21%, n = 20, Masters students; S7 Table). Exploring the professional experience of participants, >52% (n = 22) indicated they were mentors (to which group was not defined), which is a critical position of leadership for student development [8, 22, 23].

**Table 2:** Management group membership.

Response	%	n =
Yes (internal to your organization)	58.14%	25
Yes (external to your employer)	18.60%	8
No	39.53%	17
	**Total**	**43**

### Exploring participants’ understanding of leadership

**Fig 1:**
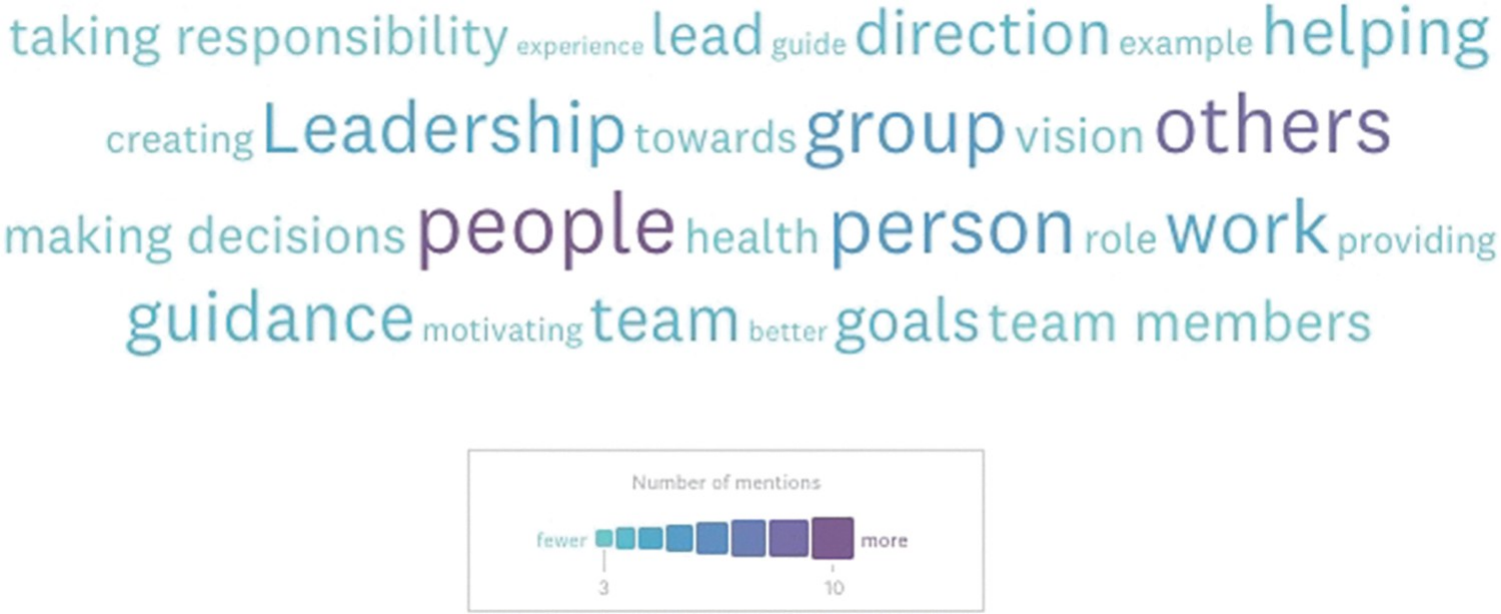
Word-cloud (word frequency) based analysis of the free-text question “What do you understand leadership to be?’ (n = 36). Exploring the most frequent (relevant) terms used in the free-text participants’ understanding of what leadership is, the top five used words/terms were: people (27%, n = 10), others (~24%, n = 9), group (~16%, n = 6), guidance (~11%, n = 4), and helping, team, direction, goals (all at ~11%, n = 4).

To profile participants’ understanding of leadership pre-survey, a free-text question was asked: “What do you understand leadership to be? (e.g., concepts, attributes)”. A semi-quantitative (word frequency) word-cloud-based analysis (n = 36) highlighted key terms such as: group, others, helping, guidance, work, guidance and team (Fig 1). Examining selected individual responses (S1 Fig), clear themes can be recognized (paraphrased/clarified), including: Ownership, accountability, responsibility (decision making); Developing and helping team members; Setting and achievement of group goals; and Communication.

**Fig 2:**
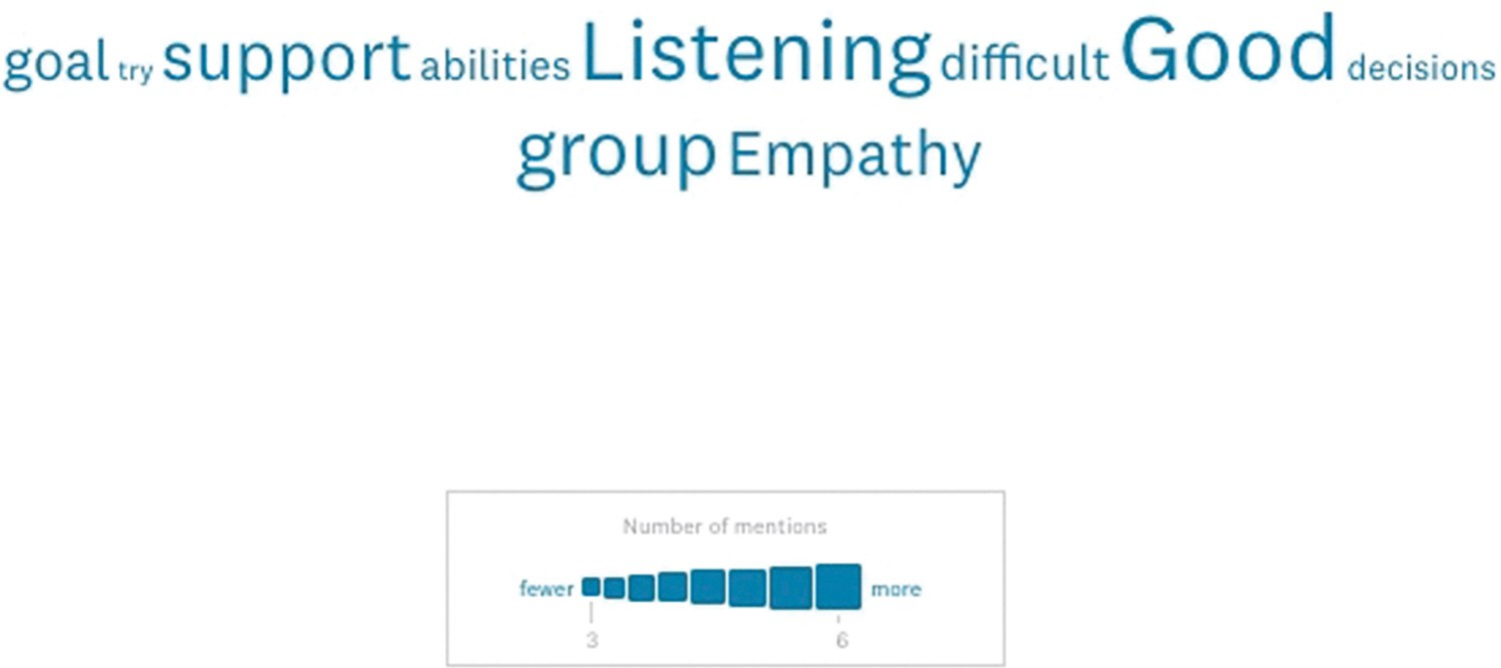
Word cloud of free-text responses to: What skills/characteristic make a good leader? Frequency of word analysis in free-text responses (n = 31).

Evaluating participants’ self-evaluation of their leadership abilities, >62% (n = 22) believed they were good leaders (Table 3, S8 Table). Investigating responses to what skills/characteristics participants thought make a good leader (n = 31), frequency of word analysis in free-text responses (Fig 2) highlighted support (~13%), listening (~16%), group (~13%), empathy (~13%), and goal (~10%) as the most common terms. ~37% (n = 13) indicated and demonstrated that they knew what EQ (emotional intelligence) was. >94% (n = 33) indicated that they believed they were honest/authentic with their colleagues. Furthermore, ~29% (n = 10) indicated that they knew what their personality type was, of which 70% (n = 7) were able demonstrate that they did indeed know their personally type (providing Myers-Briggs Type Indicator types). >68% (n = 24) agreed that they could recognize others communication style and change their approach to match.

**Table 3:** Participants self-evaluation of their leadership ability: “Am I a good leader”.

Response	%	n =
Strongly agree	8.57%	3
Agree	54.29%	19
Neither agree nor disagree	28.57%	10
Disagree	0.00%	0
Strongly disagree	0.00%	0
Not applicable.	8.57%	3
	**Total**	**35**

### Participants’ existing leadership skills/training

Exploring participants self-declared leadership training, ~38% (n = 13) stated they had received leadership training. Where given, training course details from free text responses (n = 17) indicated, EMBO leadership courses (n = 2), online leadership courses (n = 2), other leadership courses (n = 3, including n = 1 supervisory course, n = 1 MBA, n = 1 gender specific leadership course). Furthermore, >28% (n = 10) indicated they had undergone evaluation of their leadership capabilities (n = 3 both online and in-person evaluation, n = 2 online, n = 2 indicated self-reflection/reading books). ~26% (n = 9) of respondents indicated that they had been offered management training, of which ~22% (n = 7) indicated that this training was mandatory. Importantly, ~94% (n = 31) of self-identified research group leaders indicated that they had not received any formal leadership training to assist in leading their research group. Exploring self-identified management skill sets, ~29% (n = 10) indicated that they had undertaken time management training, and ~26% (n = 9) conflict resolution/management training.

Investigating leadership training in participants with formal academic titles, no senior staff (senior lecturers and all grades of professor) ~83% (n = 10) had received any. However, 25% (n = 3) had undergone management training. In contrast, 37.5% (n = 3) of lecturers had received leadership training, and 25% (n = 2) had also undergone management training.

Examining proactive planning for career development through the use of an individual development plan (IDP) across all academic categories, ~43% (n = 15) had created their own IDP, with ~14% (n = 5) using specific resources (to create their IDP).

### Leadership evaluation- Systemic and Hierarchical thinking dimensions

Previous work exploring leadership utilized the Leadership Attitudes and Beliefs Scale (LABS) tool [13], [14]. LABS is a specific and technical tool for the evaluation of leadership, with two independent areas of evaluation, Systemic and Hierarchical. The LABS Systemic Thinking dimension survey questions investigate participants attitudes to organizational leadership as individuals’ accountabilities, investigating the perceptions of the role of open communication and adaptability in leadership towards an organization’s success. The LABS Hierarchical Thinking dimension survey questions investigate hierarchical organizational leadership and stances on assigning hierarchical responsibility for success or failure [11, 14]. Furthermore, more tailored questions were written to fit the Systemic and Hierarchical categories and added to the LABS tool questions in the survey (in addition to the LABS questions) (**S2 Appendix**).

### Evaluating LABS systemic thinking dimension questions

100% (n = 15) of participants agreed: an effective organization develops its human resources; that leadership activities should lead to discussions about the future; and effective leadership involves finding resources to allow adaptation to changing conditions. >86% (n = 13) of participants agreed that individuals need to take initiative to help an organization accomplish its goals. >78% (n = 11) agreed that leadership processes involve the participation of all organization members. >93% (n = 14) agreed that leadership should encourage innovation, and >92% (n = 13) agreed that good leadership requires that ethical issues have high priority. >92% (n = 13) agreed that successful organizations make continuous learning their highest priority, and >85% (n = 12) agreed that anticipating the future is one of the most important roles of leadership processes. Interestingly, >78% (n = 11) agree that Environmental preservation should be a core value of every organization. ~93% (n = 13) agree that organizations must be ready to adapt to changes that occur outside the organization. >85% (n = 12) agreed that an organization needs flexibility in order to adapt to a rapidly changing world, and >86% (n = 13) agreed that an organization needs to be responsible for accomplishing its goals.

### Exploring leadership perceptions

Systemic thinking is associated with attitudes and beliefs that leadership is a process involving members who are working together toward a common purpose. Exploring leadership perceptions of the respondents, 80% (n = 12) strongly agreed that leadership was important (20% agreed). ~86% (n = 13) agreed that good leadership was important for their own personal development, and ~32% (n = 11) of respondents had set their own leadership goals. Importantly, ~86% (n = 30) of respondents believed that it was important to gain leadership skills and training, and ~46% (n = 11) agreed that leadership can be taught. However, only ~46% (n = 7) agreed that their line manager was a good leader, with ~32% (n = 5) disagreeing. In a more professional academic capacity, 80% (n = 12) of respondents thought it was important for lecturers to have formal leadership training, and 80% (n = 12) agreed that leadership training should be part of the postgraduate educational curriculum (in the biological sciences).

### Evaluating LABS hierarchical thinking dimension questions

Hierarchical thinking is associated with attitudes and beliefs that leadership is best in a structured, top-down manner where information flows from the top and there are clear delineations on who is a leader and who is a follower. >46% (n = 6) of participants agreed that a leader must control the group or organization, and while there was a range of responses, overall >38% (n = 5) disagreed with this statement (S9 Table). Exploring attitudes towards the belief that a leader must maintain tight control of the organization, ~23% (n = 3) agreed, while ~38% (n = 5) neither agreed or disagreed, and an equal proportion ~38% (n = 5) disagreed that they did. Interestingly, ~58% (n = 7) disagreed with “Do you believe a leader should maintain complete authority?”, whereas ~42% (n = 5) neither agreed nor disagreed. Investigating attitudes towards the roles of a leader ~68% (n = 9) of responses agreed that a leader should take charge of the group (n = 0 disagreed), and only ~30% (n = 4) agreed the main tasks of a leader were to make and communicate decisions (~23%, n = 3, disagreed; and ~46%, n = 6, neither agreed nor disagreed). There was a range of answers towards the statement “The main task of a leader is to make the important decisions for an organization”, but overall >61% (n = 8) agreed (S10 Table). Examining attitudes towards credit awarded to leaders (S11 Table), the majority of responses (>61%, n = 8) agreed that positional leaders deserved credit for the success of an organization (~15%, n = 2, disagreed). Supporting this, ~78% (n = 10) agreed that leaders of an organization are responsible for taking risks (>15%, n = 2, disagreed). Opinions were split on whether it was important for a single leader to emerge in a group, and while ~38% (n = 5) agreed, ~46% (n = 6) disagreed. ~54% (n = 7) of responses disagreed however that members should be completely loyal to leaders of an organization (interestingly ~8% agreed). >46% (n = 6) disagreed that leaders were the most important members of an organization (in contrast >15%, n = 2, agreed). Overall >46% (n = 6) agreed that Leaders are responsible for the security of organization members, with only >15 (n = 2) disagreeing. 85% (n = 11) agreed that “an organization should try to remain as stable as possible” (n = 0 disagreeing). Attitudes towards getting new leaders to fix an organization in danger of failure favored those that agreed >38% (n = 5), however ~23% (n = 3) disagreed with this (S12 Table).

### Exploring leadership attitudes

Investigating the value of leadership, 100% (n = 13) of respondents agreed (~77%, n = 10, strongly) that they valued good leadership. ~84% (n = 11) agreed that good leadership is important for their managers, of which 63% (n = 7) agreed strongly. Interestingly, in agreement with previous responses, where ~65% (n = 22) believed they are a good leader (S4 Table), here >68% (n = 9) believed it was likely they were good leaders. This is interesting when combined with the >68% (n = 9) who believe it likely are effective leaders (only n = 1 thought they were “very likely” an effective leader) (Table 4).

**Table 4:** Do you believe you are an effective leader?

Response	%	n =
Very likely	7.69%	1
Likely	61.54%	8
Neither likely nor unlikely	23.08%	3
Unlikely	7.69%	1
Very unlikely	0%	0
	**Total**	**13**

Exploring free text responses of how participants believed they demonstrated effective leadership, two key areas were categorised (summarizing): their staff doing well (achieving their goals), and performance metrics/feedback. Probing participants attitudes towards aiming to improve their leadership knowledge and skills revealed that ~77% (n = 10) of respondents believe they should improve their leadership attributes (answers split equally between “A great deal” and “A lot”) (Table 5).

**Table 5:** Should you aim to improve your leadership knowledge and skills?

Response	%	n =
A great deal	38.46%	5
A lot	38.46%	5
A moderate amount	23.08%	3
A little	0%	0
None at all	0%	0
	**Total**	**13**

Furthermore, ~77% (n = 10) indicated observing leadership positions did influence their own leadership development (~56% indicated “A Lot”) (Table 6). This attitude was supported further by ~84% (n = 11) of responses that indicated practicing leadership skills very positivity influenced their own leadership development (split almost equally between “A lot” or”A great deal”) (Table 7).

**Table 6:** How much influence does observe others in leadership positions have on your own leadership development?

Response	%	n =
A great deal	23.08%	3
A lot	53.85%	7
A moderate amount	15.38%	2
A little	7.69%	1
None at all	0.00%	0
	**Total**	13

**Table 7:** How much influence does practicing particular leadership skills yourself have on your own leadership development?

Response	%	n =
A great deal	46.15%	6
A lot	38.46%	5
A moderate amount	0.00%	0
A little	15.38%	2
None at all	0.00%	0
	**Total**	**13**

### Evaluation of Systemic v Hierarchical thinking

**Fig 3:**
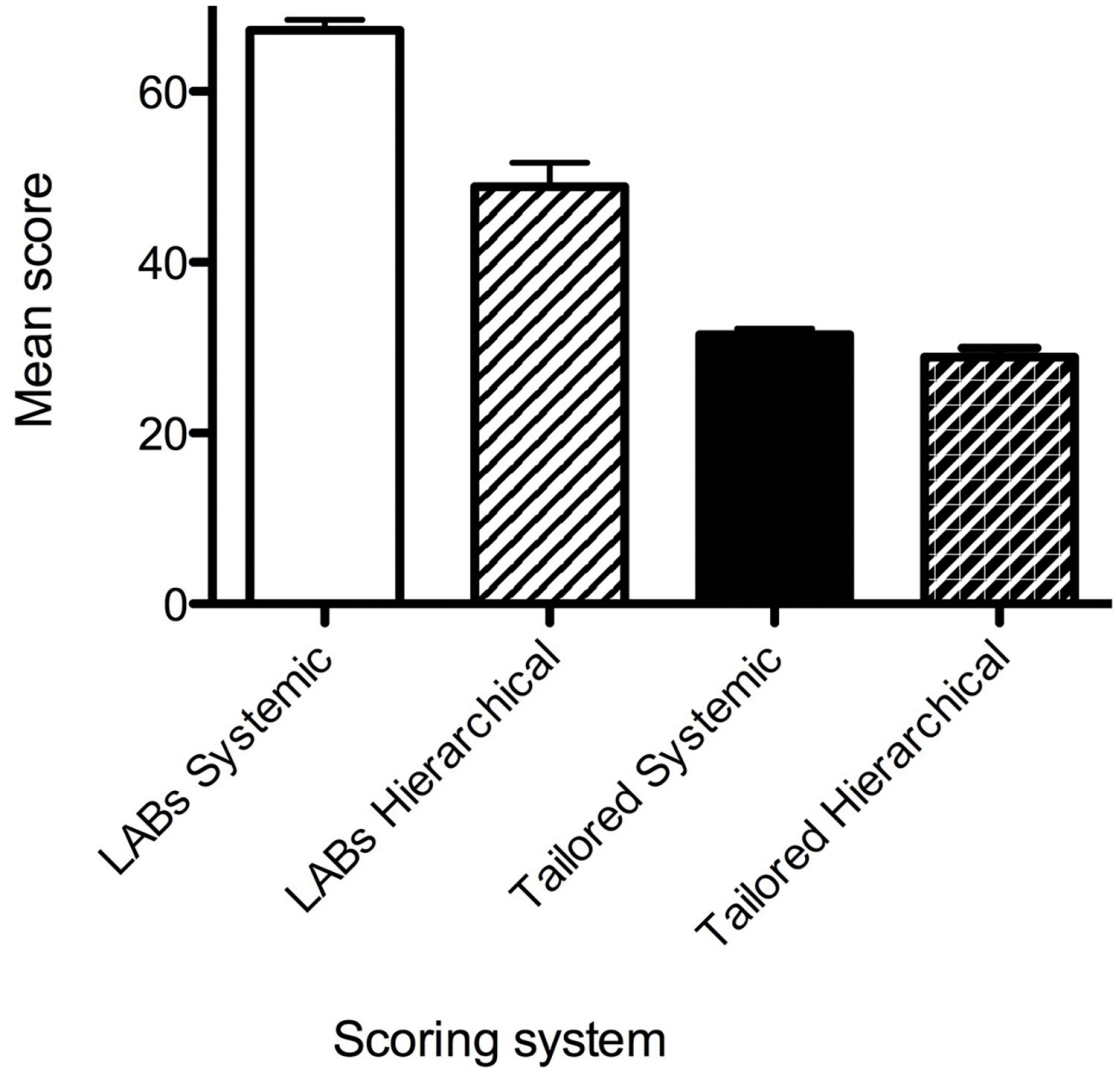
Comparison of scoring Systemic or Hierarchical thinking questions (LABS v Tailored Questions). Mean score and SD shown. Calculated as per [19]).

Overall, the study participants were found to be more oriented to the Systemic Thinking dimensions (mean 67.1, SD 4.7), compared to the Hierarchical Thinking dimensions (mean 45.4, SD 10.2) using LABS scoring (Table 8) (Fig 3) [19].

**Table 8:** Scoring Systemic v Hierarchical thinking questions.

	LABS scoring		Tailored Questions	
**Category**	Mean score	SD	Mean score	SD
Systemic Thinking	67.1	4.7	33.8	2.7
Hierarchical Thinking	45.4	10.2	26.9	3.75

While there was a significant difference (One-way ANOVA Kruskal-Wallis test with a Dunns post-test) between the absolute values of the LABS and Tailored Hierarchical (p>0.05), and the LABS and Tailored Systemic (p>0.005), this was due to the different questionnaires. Importantly, the Tailored Questions scores followed the same trend as the established LABS scoring system (Table 8). Again, study participants were found to be more oriented to the Systemic Thinking dimension (Perceptions) with a mean score of 33.8 (SD 2.7), compared to Hierarchical Thinking (Attitudes) with a mean score of 26.9 (SD 3.75).

Evaluating the scores (Spearman’s non-parametric correlation, two-tailed test), the only significant correlations were found in the LABS Hierarchical Thinking questions: “Do you believe a leader should maintain complete authority” compared to “Do you believe a leader should take charge of the group” (p>0.05); “Do you believe a leader should maintain complete authority” compared to “The responsibility for taking risks lies with the leaders of an organization” (p>0.05); “Do you believe a leader should maintain complete authority” compared to “The responsibility for taking risks lies with the leaders of an organization” (p>0.005); “Members should be completely loyal to the designated leaders of an organization” compared to “The responsibility for taking risks lies with the leaders of an organization” (p>0.05); “The most important members of an organization are its leaders” compared to “The responsibility for taking risks lies with the leaders of an organization” (p>0.05).

### Exploring participants’ concluding thoughts on leadership or leadership training in the biological sciences

A word-cloud word-frequency analysis on the free-text question responses to “additional thoughts/comments about the importance of leadership or leadership training in the biological sciences?” (n = 8) showed the most common terms used (excluding leadership, leadership skills, and training) were: Research 62.5% (n = 5); Career 50% (n = 4); and equally Level, Think, People (each 37.5%, n = 3) (S2 Fig).

## Discussion

While leadership skills and training are valued and evaluated in many fields, to date little is known about the state of current leadership skills in the biological sciences. Here, building on previous studies investigating leadership [16, 24, 25], “core” aspects in and around leadership in the biological sciences was profiled. This profiling captured and evaluated professionals understanding of leadership, the current state of leadership training, and their aspiration for leadership training.

The aspirations defined here, of biological sciences staff to acquire and build leadership skills, support broader findings on the need to improve leadership in higher education [26–28]. Importantly, it has been noted that academic leadership (in universities) face unique problems not seen in business or government, primarily the need for academic leaders to continue practicing their “core roles” (research and scholarship, teaching) as well as developing their own and others leadership skills [29]. This is supported here, where >50% of academic participants also managed a research group. Participants’ understanding of leadership, orientated to Systemic Thinking dimensions (or collective), has been proposed as the style most likely to produce long-term sustainability [19], important for building leadership within universities. These results suggest the biological sciences staff aspire to develop a more collaborative leadership structure, as opposed to the existing traditional hierarchical structures [30]. Strikingly, ~94% of research group leaders had not received any formal leadership training, and that the majority (>80% of participants’) thought that being able to use leadership skills would be a very positive way to influence their leadership development. This view is strongly supported by educational programs, and the research, carried out in the medical faculty, where the focus has long been on producing leaders [17, 31, 32]. Without specific leadership training, it is difficult to believe that management training will actually produce meaningful, leadership-skills focused outcomes [33, 34].

Exploring the finding centered on the Systemic Thinking dimensions, it is interesting to see that ~50% of respondents thought their line manager was a good leader. This could be interpreted to mean that an underlying issue is leaders who do not work towards collective decisions. As less than half believed that leadership can be taught, this reveals a clear need and opportunity to implement leadership training in the biological sciences’ academic environment. Improving rates of (specific) leadership training, and by extension skills and practice, would therefore likely have a significant and positive impact in these environments. This leadership training would also have the added benefit of producing a workforce with the skills to adapt to changing environments, as has been witnessed with the current worldwide COVID disruption to the higher education sector [35]. Furthermore, participants felt that leadership should encourage innovation, a view which is supported [36, 37].

Finally, it was clear from the responses that leadership skills are highly valued in the biological sciences. However, this is severely tempered by a clear understanding that the current staff lack the necessary skills to practice (or teach) leadership themselves. This is demonstrated by the clear lack of leadership training available. Until the biological sciences places a real measurable value on leadership, by incentivizing training and practice while monitoring it, this group will continue to underperform without fully training its workforce for the roles of the position (perpetuating the cycle) [38]. Improving leadership skills in teaching and training staff will have the flow on effect of improving the levels and visibility of good leadership available to undergraduate and postgraduate students, as has been seen in the medical faculty [16–18, 31, 39]. This improves students’ skill sets and satisfaction, and better prepares them as professionals for the workforce in an ever-changing world.

While a strength of this study was the international cohort (encompassing a representative spectrum of age, roles, and experiences) in the biological sciences, a limitation of this work is the cohort size (13–39 responses to individual questions). As the first study explicitly investigating leadership in the biological sciences, it is a foundation in which to build future more detailed studies investigating leadership (particularly of research group leaders). The biological sciences will develop their tailored own leadership programmes, which will support further positive long-term organizational changes (building on the current successes of equality programmes) [40, 41].

## Conclusions

These findings clearly demonstrate that staff in the biological sciences understand what leadership is, highly value good leadership, and importantly want to become better leaders by acquiring specific leadership training (and skills). Encouragingly for the development of leadership in the biological sciences, this work revealed that the skills and characteristics most believed to make you a good leader included support, listening, and empathy. These are characteristics intrinsic to transformational leaders who uplift, inspire and support co-workers, promoting/embodying values that enhance their professional environment [36, 38, 42]. The leadership aspirations of life science academics described here support the growing evidence that universities must do more to develop their leaders, through specific leadership training [38]. This will require a tailored leadership development pipeline for the biological sciences to be created (as has proven effective in medical education), to address the unique environment and specific needs of those in the biological sciences. Empowering staff with these enhanced leadership skills and abilities will clearly benefit the organization, staff and students.

## Supporting information

S1 Fig Selected individual free-text responses to: “What do you understand leadership to be?” (JPG)

S2 Fig Word-cloud of free-text answers to: “What skills/characteristics make you a good leader?” (JPG)

S1 Table Participant gender. (JPG)

S2 Table Participant age bracket. (JPG)

S3 Table Participant job description/role. (JPG)

S4 Table Participant years post PhD. (JPG)

S5 Table Participants geographic region. (JPG)

S6 Table Participants leading a research group. (JPG)

S7 Table Participants research group composition. (JPG)

S8 Table ‘Do you believe you are a good leader?’ (JPG)

S9 Table ‘Do you believe a leader must control the group or organization.’ (JPG)

S10 Table The main task of a leader is to make the important decisions for an organization. (JPG)

S11 Table Positional leaders deserve credit for the success of an organization. (JPG)

S12 Table When an organization is in danger of failure, new leaders are needed to fix its problems. (JPG)

S1 Appendix Complete survey forms and questionnaire. (PDF)

S2 Appendix Categorization (Hierarchal or Systemic) of survey instrument questions. (PDF)

## Abbreviations

LAB: Leadership Attitudes and Belief
PI: Principal Investigator
IDP: individual development plan
STEMM: Science, Technology, Engineering, Mathematics, Medicine
