# Supplementary figures and images for: Profiling leadership: Attitudes, knowledge and training in the biological sciences
Source: PLoS One. 2023 Jun 7;18(6):e0286826. doi: 10.1371/journal.pone.0286826 (PMC10246786; doi:10.1371/journal.pone.0286826)

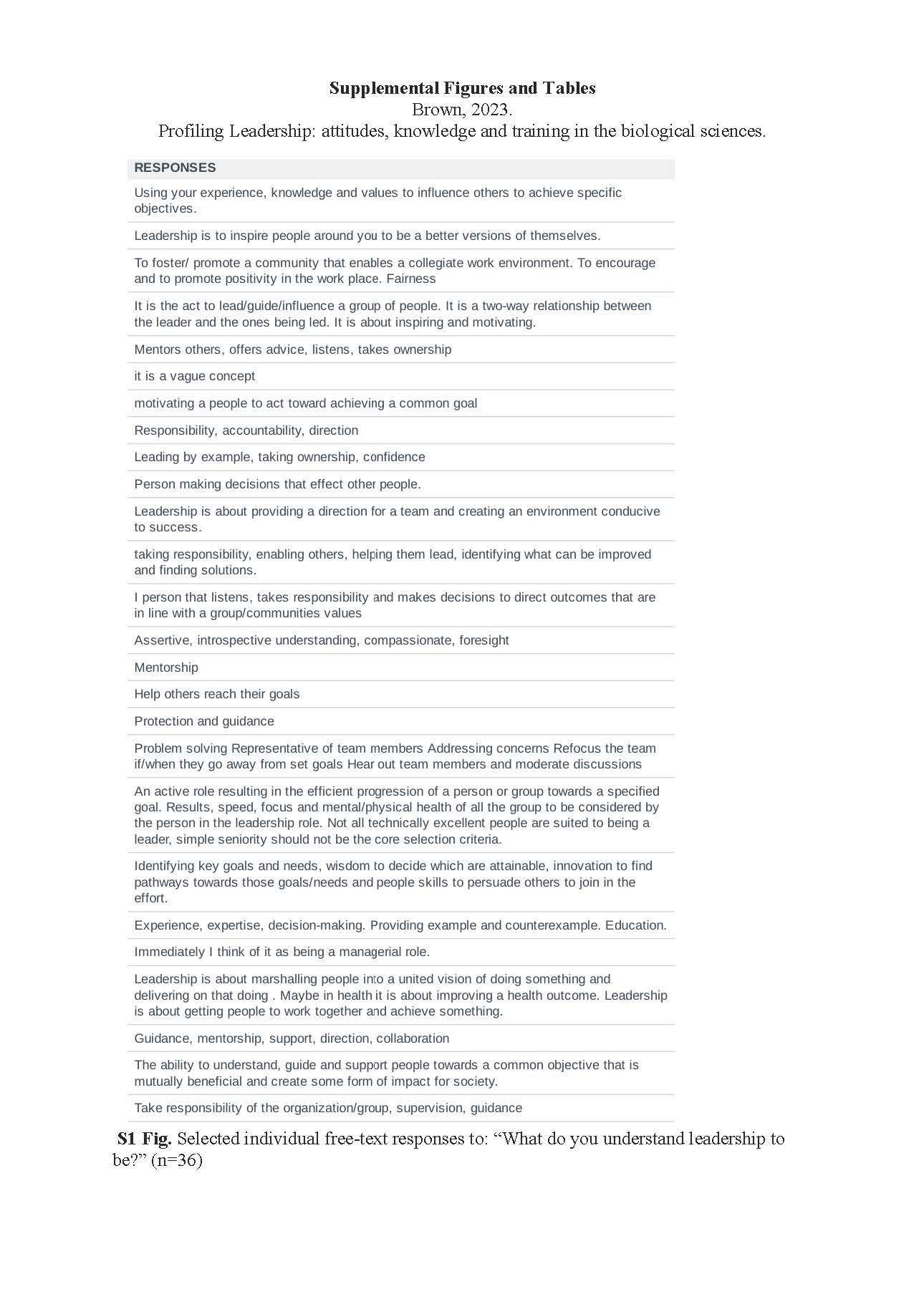

Supplement: S1 Fig — (JPG) [file pone.0286826.s001.jpg]

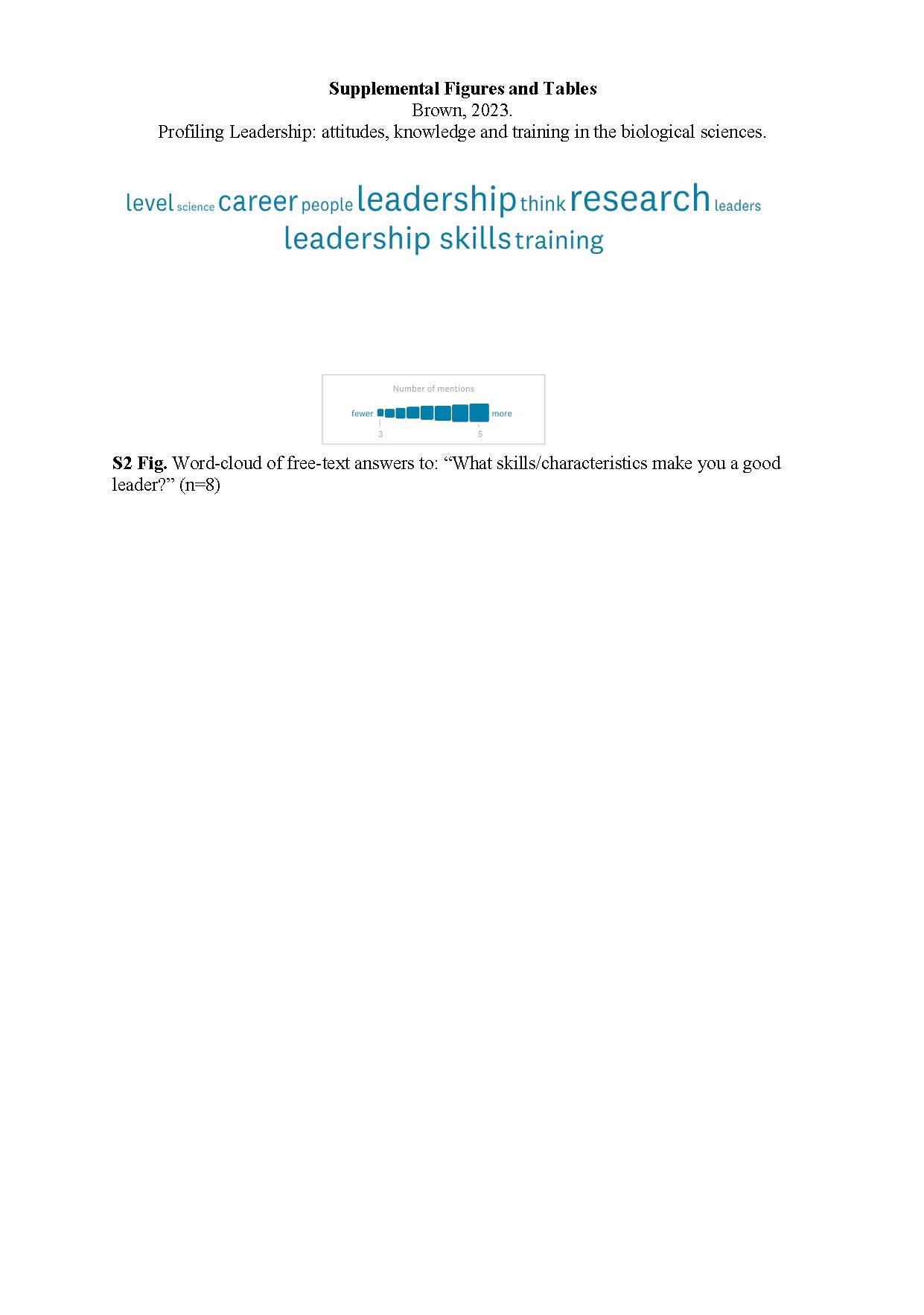

Supplement: S2 Fig — (JPG) [file pone.0286826.s002.jpg]

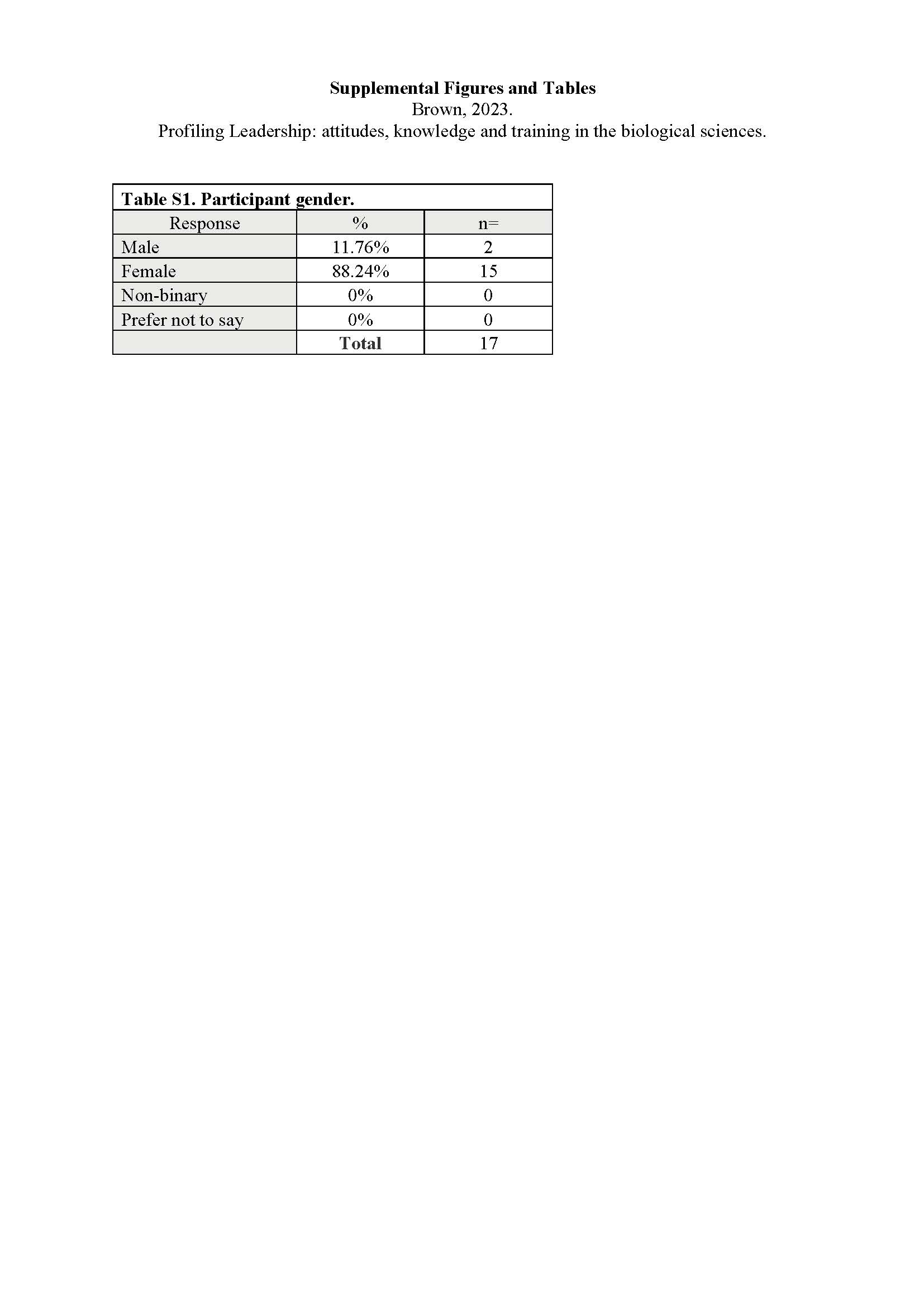

Supplement: S1 Table — (JPG) [file pone.0286826.s003.jpg]

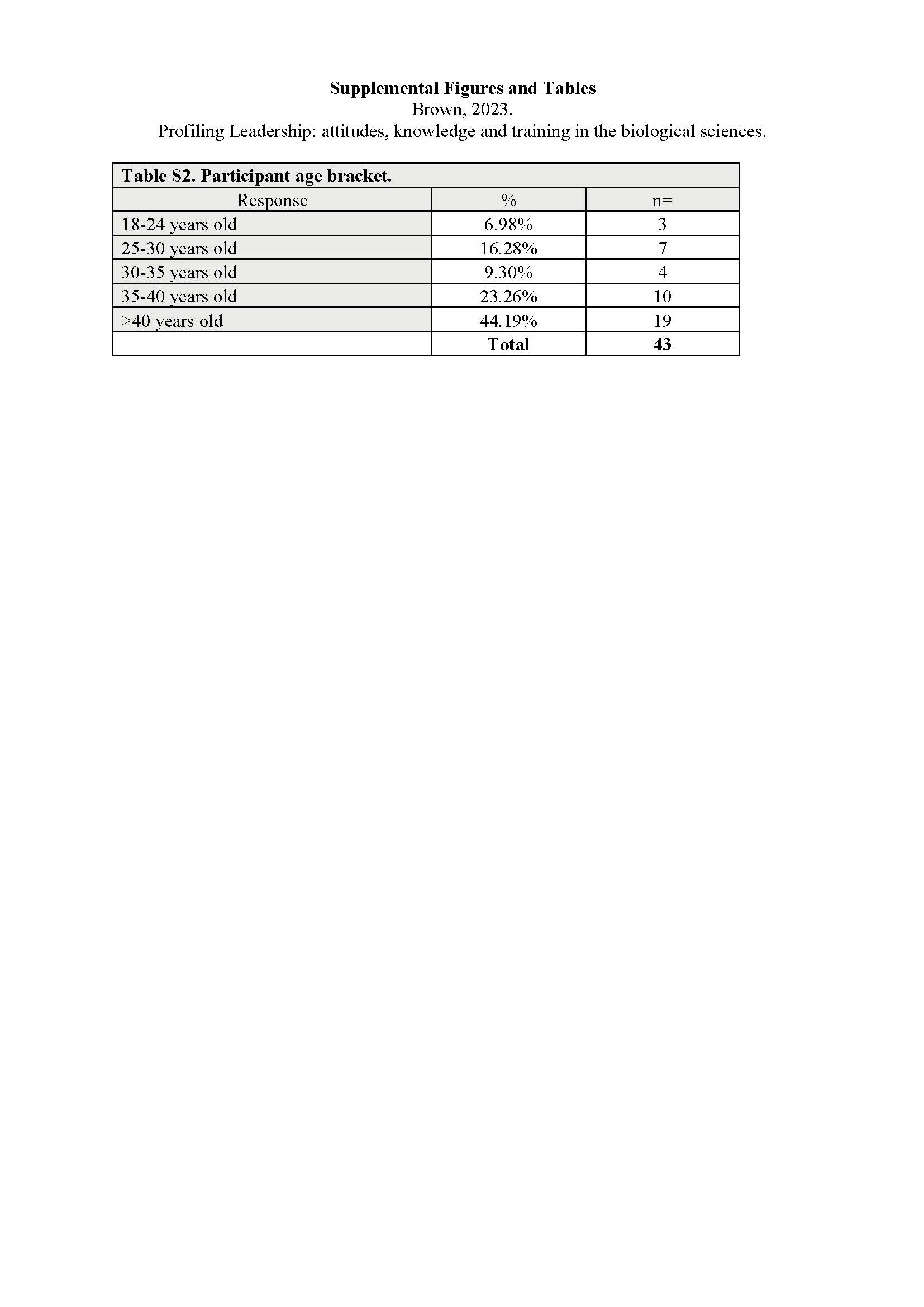

Supplement: S2 Table — (JPG) [file pone.0286826.s004.jpg]

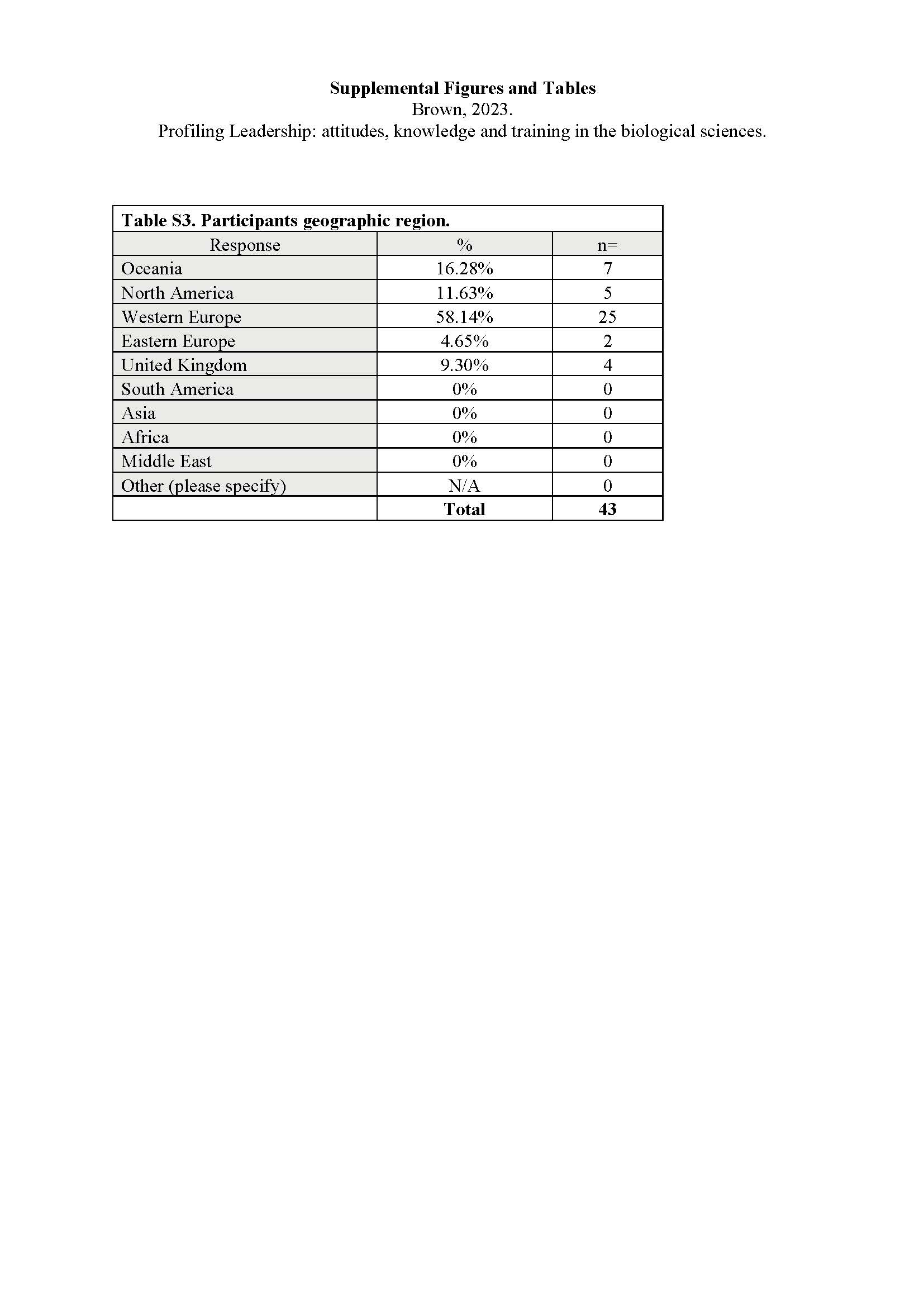

Supplement: S3 Table — (JPG) [file pone.0286826.s005.jpg]

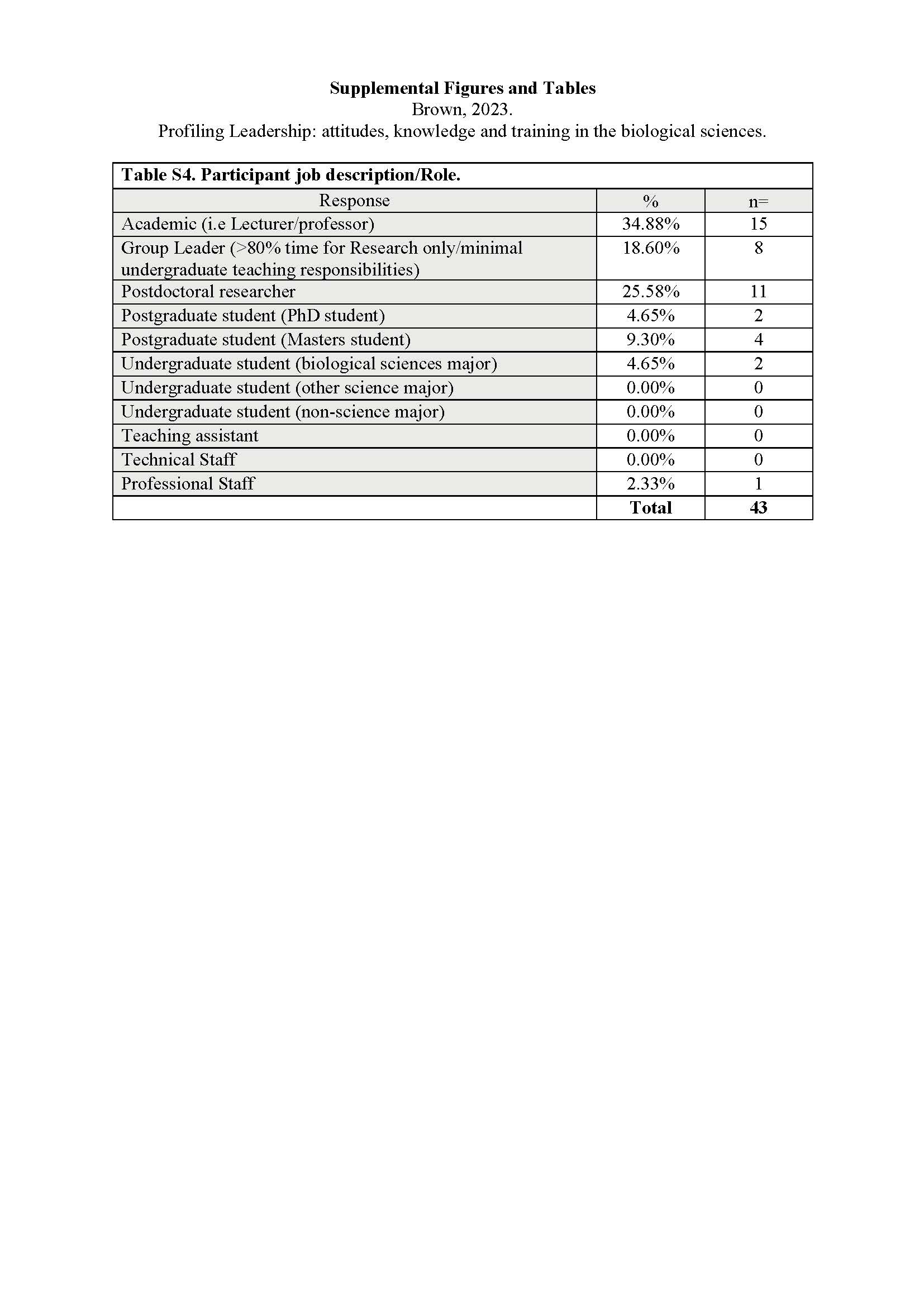

Supplement: S4 Table — (JPG) [file pone.0286826.s006.jpg]

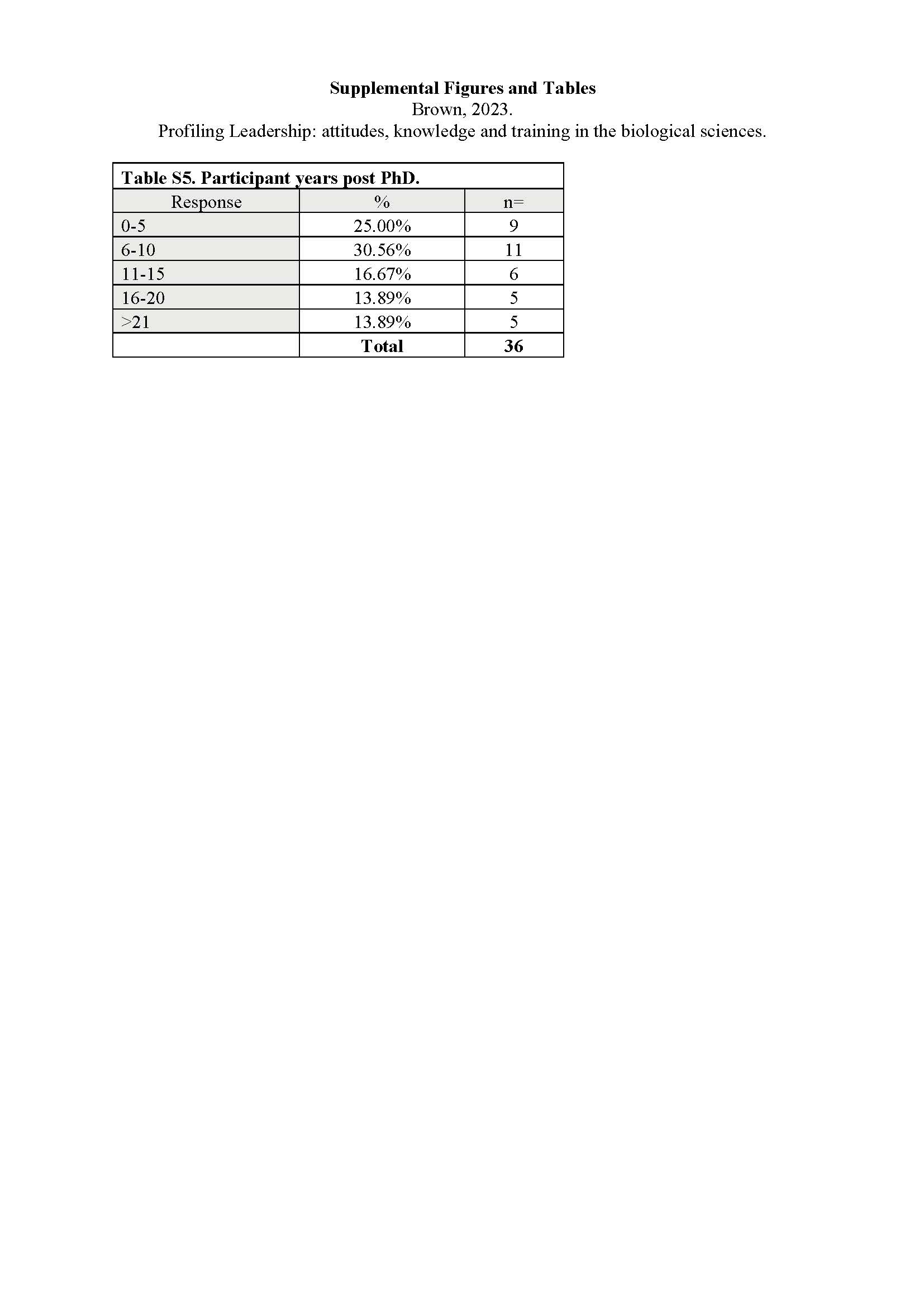

Supplement: S5 Table — (JPG) [file pone.0286826.s007.jpg]

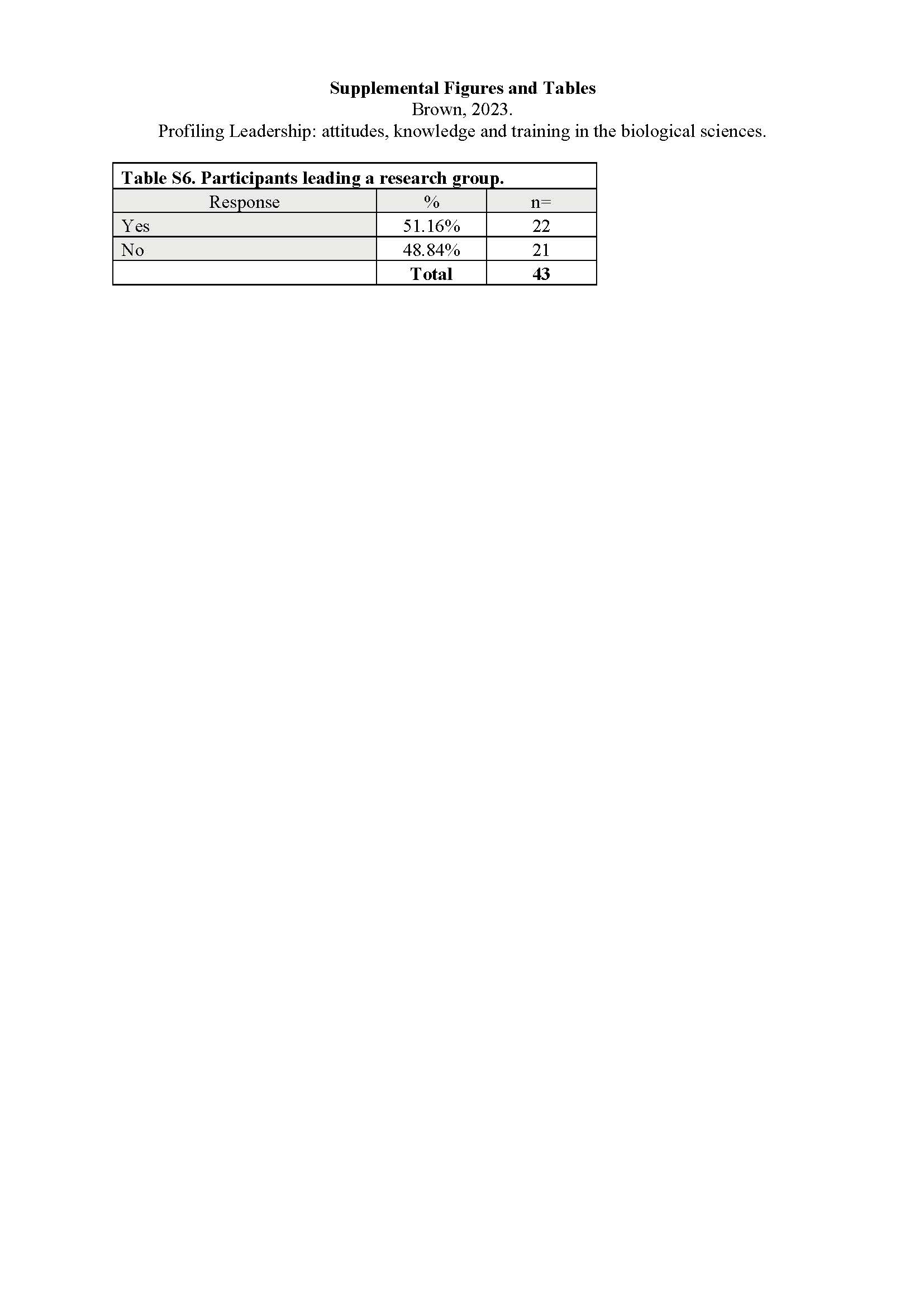

Supplement: S6 Table — (JPG) [file pone.0286826.s008.jpg]

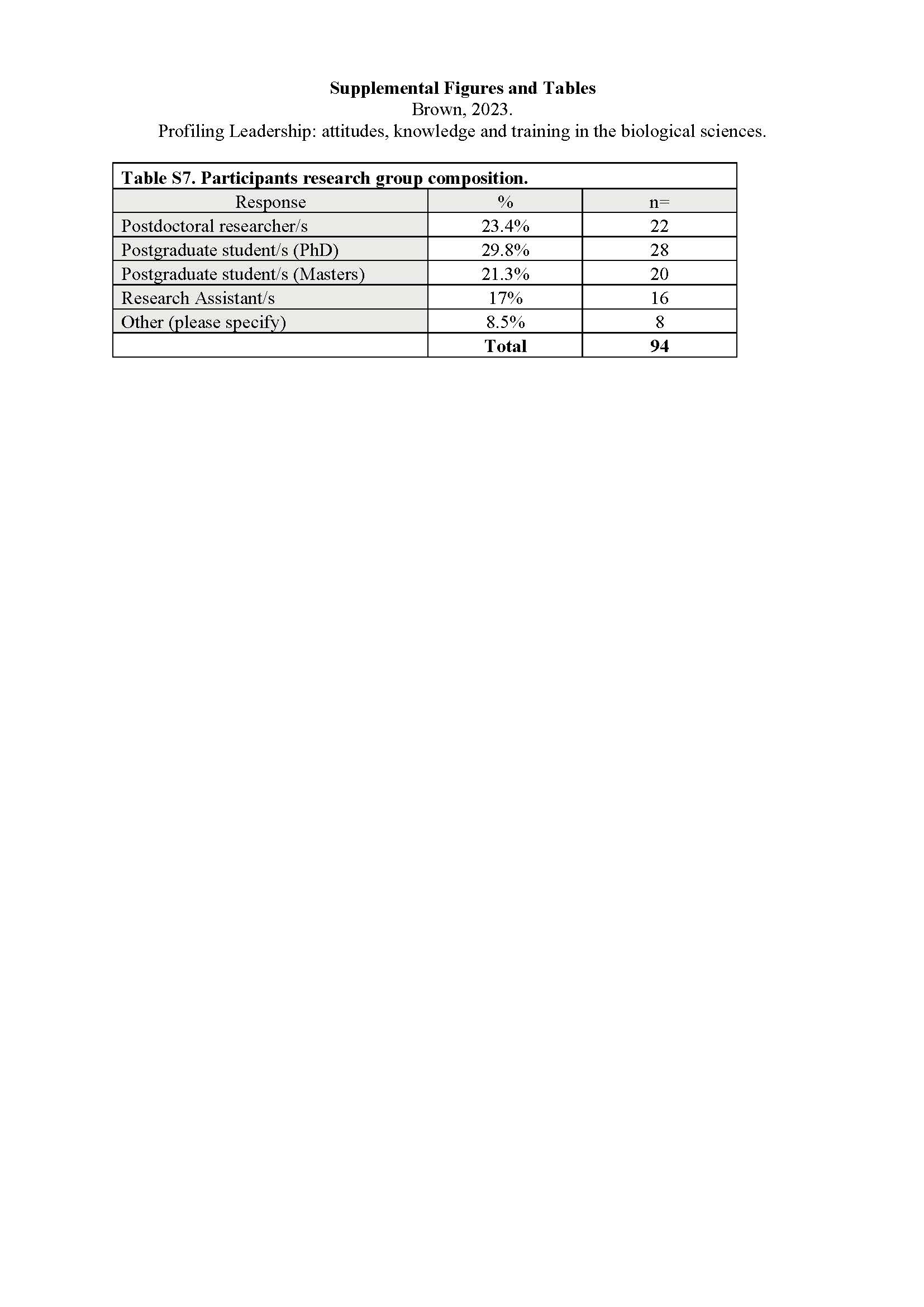

Supplement: S7 Table — (JPG) [file pone.0286826.s009.jpg]

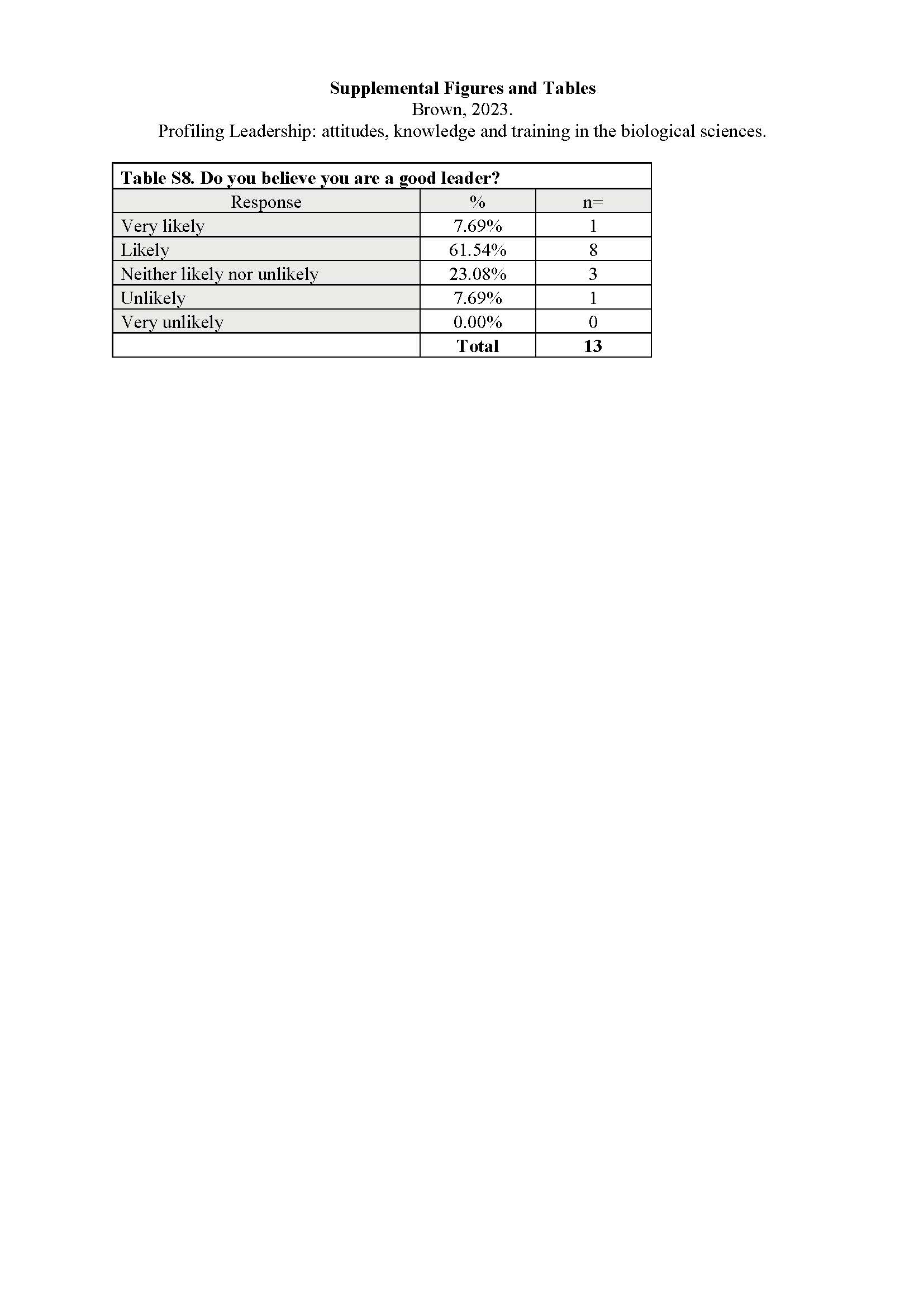

Supplement: S8 Table — (JPG) [file pone.0286826.s010.jpg]

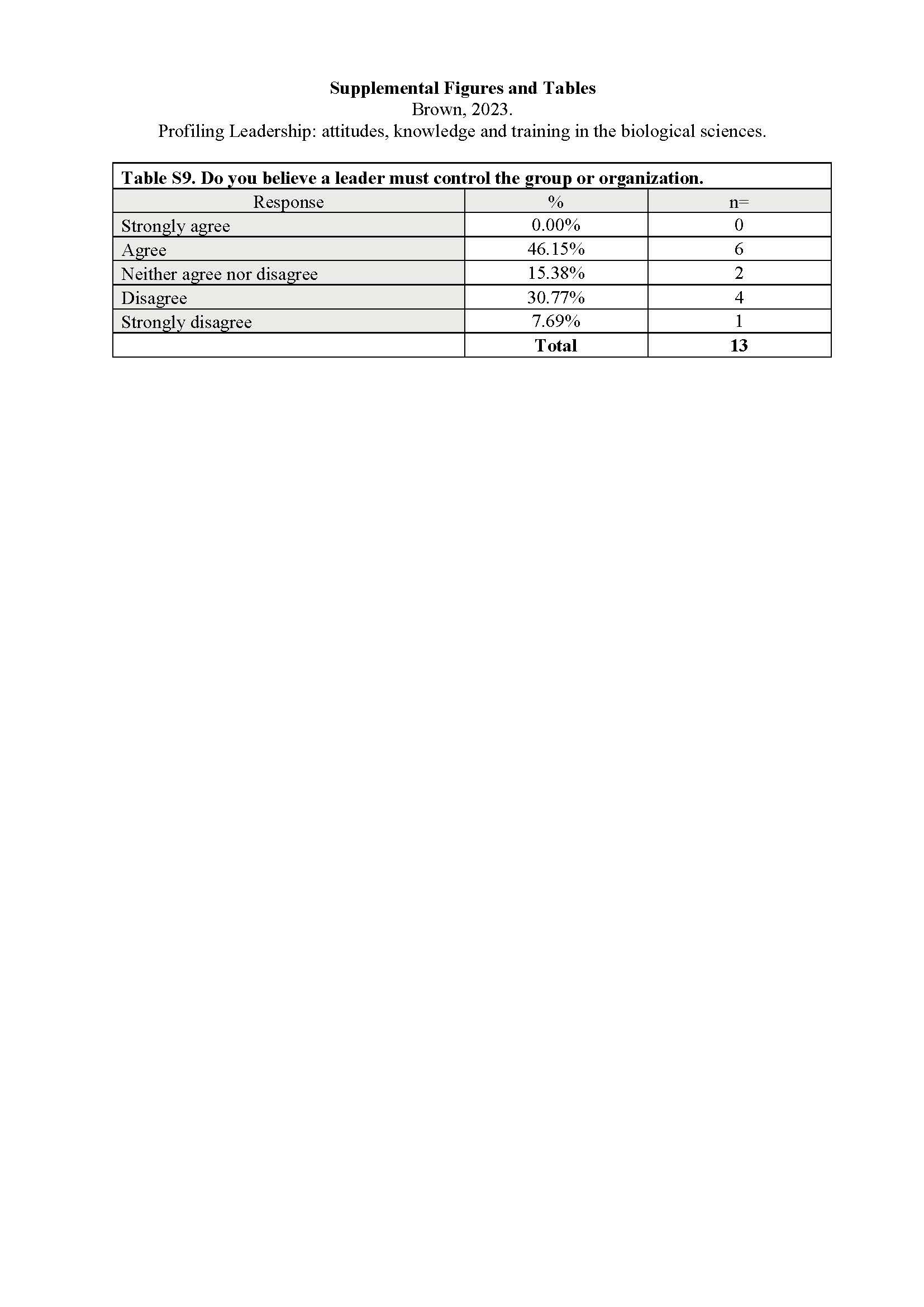

Supplement: S9 Table — (JPG) [file pone.0286826.s011.jpg]

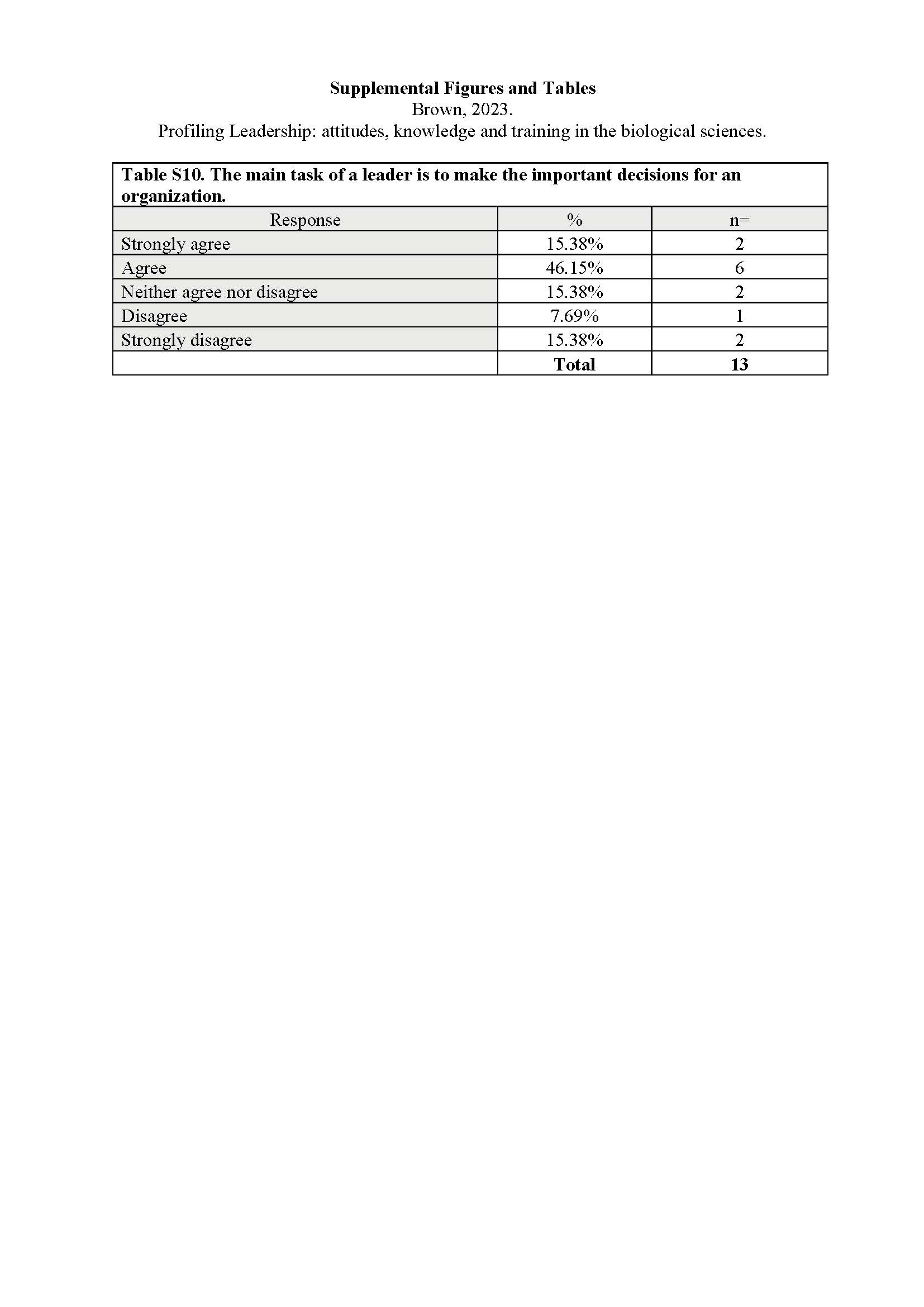

Supplement: S10 Table — (JPG) [file pone.0286826.s012.jpg]

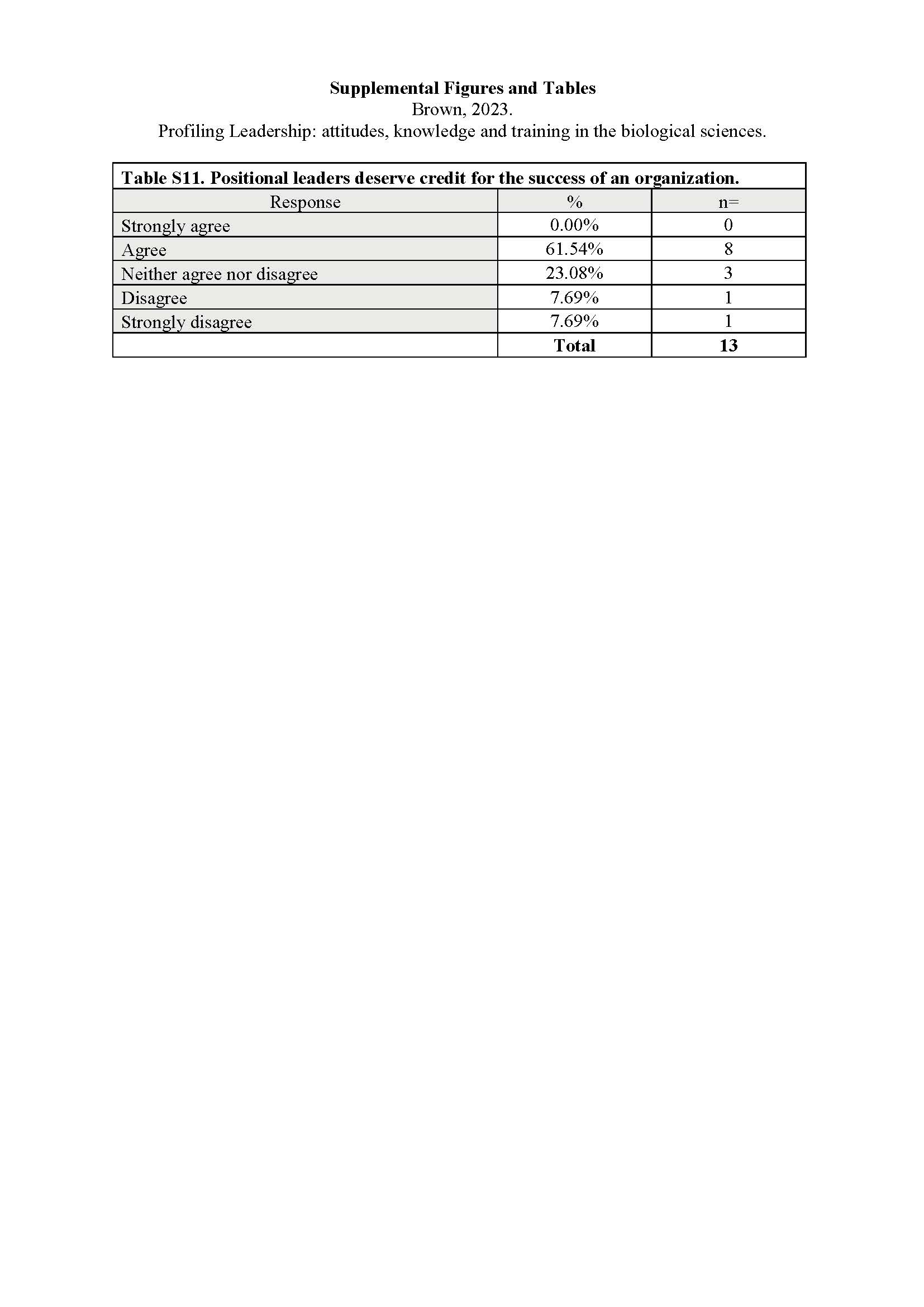

Supplement: S11 Table — (JPG) [file pone.0286826.s013.jpg]

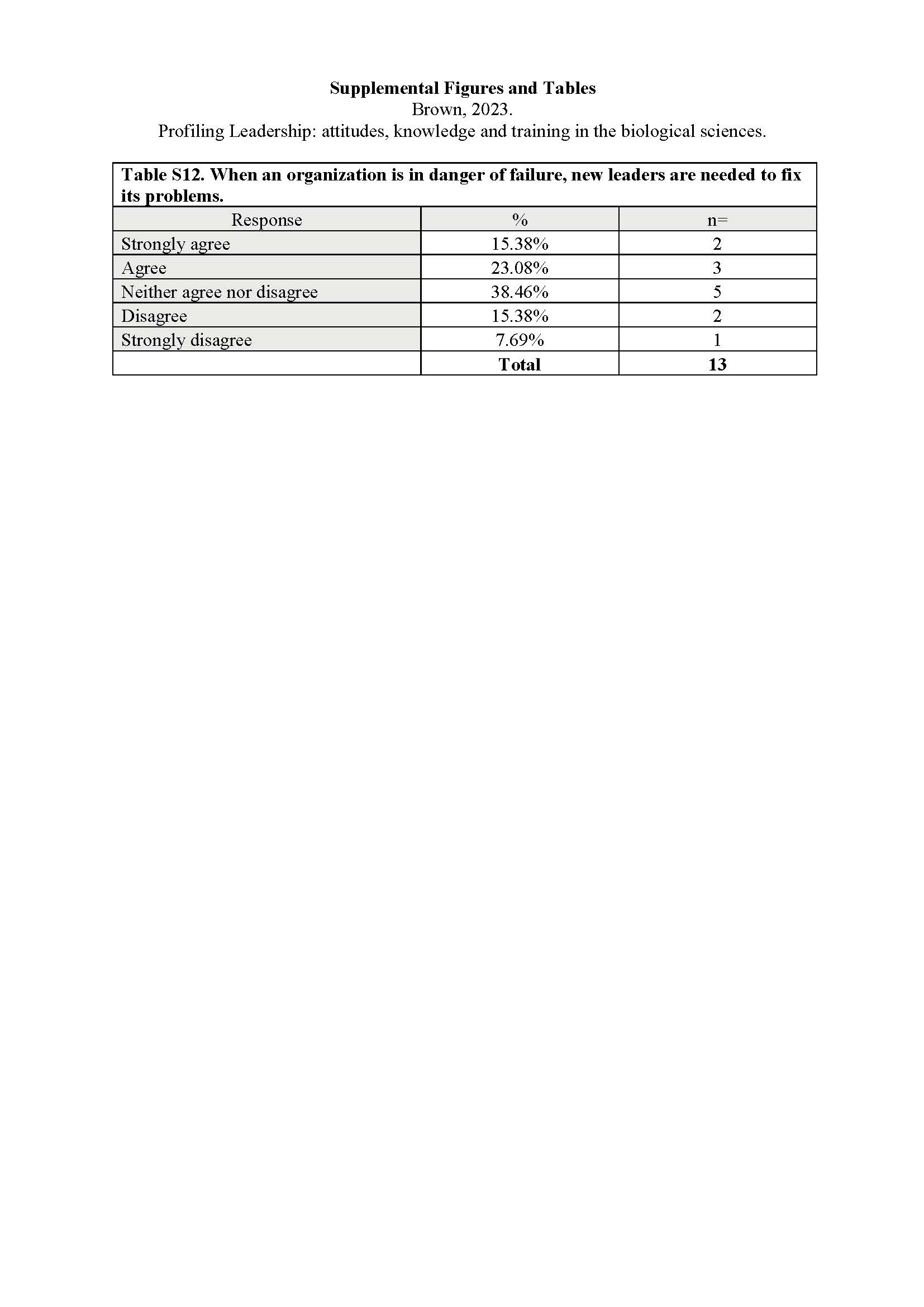

Supplement: S12 Table — (JPG) [file pone.0286826.s014.jpg]
