# Supplementary material for: Profiling leadership: Attitudes, knowledge and training in the biological sciences
Source: PLoS One. 2023 Jun 7;18(6):e0286826. doi: 10.1371/journal.pone.0286826 (PMC10246786; doi:10.1371/journal.pone.0286826)
Supplement: S1 Appendix — (PDF) [file pone.0286826.s015.pdf]

Appendix 1

Leadership survey

1. Biological Sciences Leadership survey

Dear Participant,

My name is James Brown and I am undertaking research at the University of Limerick. I am investigating and profiling different aspects of leadership, as seen and experienced in the biological sciences. The aim of this research is to capture and evaluate key data (including attitudes, training, knowledge and skills) about leadership for those in the biological sciences (including academic staff, researchers and postgraduate students).

Your participation is voluntary, and you have the right to withdraw at any time. Any responses given are anonymous and non-identifiable. To participate in this study, you must be between the ages of 18 and 65.

If you have further questions regarding this research, please feel free to get in touch with myself (james.brown@ul.ie), or the address below.

If you have concerns about this study and wish to contact someone independent, you may contact: The Chair, Faculty of Science & Engineering Research Ethics Committee, University of Limerick, Limerick. Tel: 061 237719

Yours sincerely,

James Brown

This project has University of Limerick ethics approval.

* Ethical Consent Form

I declare that I am willing to take part in research for the project entitled Assessing Leadership skills attitudes and knowledge in the biological sciences.

· I declare that I have been fully briefed on the nature of this study and my role in it and have been given the opportunity to ask questions before agreeing to participate.

· The nature of my participation has been explained to me, and I have full knowledge of how the information collected will be used.

· I am aware that such information may also be used in future academic presentations and publications about this study.

· I fully understand that there is no obligation on me to participate in this study.

· I fully understand that I am free to withdraw my participation without having to explain or give a reason, up to a period of two weeks after the data collection is completed.

· I acknowledge that the researcher does guarantee that they will not use my name or any other information, that would identify me in any outputs of the research.

· I declare that I have read and fully understand the contents of the Research Privacy Notice.

- ◯ Yes
- ◯ No

2. Survey Directions

This voluntary, anonymous, questionnaire has 4 sections (total time approx. 20 min):

1. **Demographics**
2. **Your Leadership training, skills and knowledge**
3. **Your Perceptions of Leadership**
4. **Your Leadership attitudes**

Voluntary participation in this survey does not constitute authorship.

It is intended that the results from this work will be submitted for peer-review in an open access publication (with a Creative Commons license BY CC), and will be deposited/available for comment in a preprint service where possible.

* What do you understand leadership to be? (eg concepts, attributes)

3. Section 1 (of 4): Participant demographics (eg Position/Role, gender, location)

* What is your job description/Role

- Academic (i.e Lecturer/professor)
- Group Leader (>80% time for Research only/minimal undergraduate teaching responsibilities)
- Postdoctoral researcher
- Postgraduate student (PhD student)
- Postgraduate student (Masters student)
- Undergraduate student (biological sciences major)
- Undergraduate student (other science major)
- Undergraduate student (non-science major)
- Teaching assistant
- Technical Staff
- Professional Staff

* If you are an academic or post PhD, what is your title (or local equivalent: https://en.wikipedia.org/wiki/List_of_academic_ranks):

- Postdoctoral Researcher
- Lecturer
- Senior Lecturer
- Professor
- Other (please specify)

* Are you part of any management groups (i.e. committees, councils)?

- [ ] Yes (internal to your organisation)
- [ ] Yes (external to your employer)
- [ ] No

* If you are post PhD, how many years ago did you receive your doctorate?

- 0-5
- 6-10
- 11-15
- 16-20
- >21

* What geographic area do you work in?

- Oceania
- North America
- Western Europe
- Eastern Europe
- United Kingdom
- South America
- Asia
- Africa
- Middle East

Other (please specify)

* Do you manage a research group?

- Yes
- No

* What is the size and composition of your group

- Postdoctoral researcher/s
- Postgraduate student/s (PhD)
- Postgraduate student/s (Masters)
- Research Assistant/s

Other (please specify)

* Do you supervise undergraduate students (eg final year projects; either formally or informally)?

- Yes
- No

* What is your age bracket?

- 18-24 years old
- 25-30 years old
- 30-35 years old
- 35-40 years old
- >40 years old
- Prefer not to say.

* Are you mentor?

- Yes
- No

* What is your gender

- ◯ Male
- ◯ Female
- ◯ non-binary
- ◯ Prefer not to say

4. Section 2 (of 4): Your leadership Skills, Training and knowledge.

* Have you received any leadership training?

Yes

No

If you are willing please provide details (i.e topic, online, in person)?

* Have you evaluated your leadership capabilities?

Yes

No

If willing please provide details (i.e online, in person)?

* Have you been offered any management training?

Yes

No

* Was this training mandatory?

Yes

No

* If you are a research group leader, have you received any specific training related to leading your research group (excluding financial systems and administrative training)?

Yes

No

What was the training?

* Have you undergone any time management training?

Yes

No

* Have you created an individual development plan?

- Yes
- No

Did you use any specific resources (eg online tools)?

- Yes
- No

Please specify

* Have you undergone any conflict resolution/management training?

- Yes
- No

* If you are in any leadership positions, do you believe you are a good leader?

- Strongly agree
- Agree
- Neither agree nor disagree
- Disagree
- Strongly disagree
- Not applicabile.

* What skills/characteristics make you a good leader?

* Do you know your personality type?

- Yes
- No

What is your personality type?

* Do you know what EQ is?

- Yes
- No

What is EQ?

* Have you set any leadership goals?

- Yes
- No

* Are you honest/authentic in your dealing with colleagues in the workplace?

- Strongly agree
- Agree
- Neither agree nor disagree
- Disagree
- Strongly disagree

* You able to recognize other's communication styles, & approach them effectively based on their style?

- Strongly agree
- Agree
- Neither agree nor disagree
- Disagree
- Strongly disagree

* Do you believe it important for you to gain leadership skills/training?

- Yes
- No

5. Section 3 (of 4): Perceptions of leadership

* Do you think leadership is important?

- Strongly agree
- Agree
- Neither agree nor disagree
- Disagree
- Strongly disagree

* Do you think having formal leadership training/skills is important for lecturers?

- Strongly agree
- Agree
- Neither agree nor disagree
- Disagree
- Strongly disagree

* Do you think having formal leadership training/skills is important for research group leaders?

- Strongly agree
- Agree
- Neither agree nor disagree
- Disagree
- Strongly disagree

* Should formal leadership training/skills be part of postgraduate education (in the biological sciences)?

- Strongly agree
- Agree
- Neither agree nor disagree
- Disagree
- Strongly disagree

* Is having good leadership (in your workplace) important for your personal development?

- Strongly agree
- Agree
- Neither agree nor disagree
- Disagree
- Strongly disagree

* Do you believe your line manager/supervisor is a good leader?

- Strongly agree
- Agree
- Neither agree nor disagree
- Disagree
- Strongly disagree

* Do you think leadership is something that can be taught?

- Strongly agree
- Agree
- Neither agree nor disagree
- Disagree
- Strongly disagree

* An effective organization develops its human resources.

- Strongly agree
- Agree
- Neither agree nor disagree
- Disagree
- Strongly disagree

* Leadership activities should foster discussions about the future.

- Strongly agree
- Agree
- Neither agree nor disagree
- Disagree
- Strongly disagree

* Effective leadership seeks out resources needed to adapt to a changing world.

- Strongly agree
- Agree
- Neither agree nor disagree
- Disagree
- Strongly disagree

* Individuals need to take initiative to help their organization accomplish its goals.

- Strongly agree
- Agree
- Neither agree nor disagree
- Disagree
- Strongly disagree

* Leadership should encourage innovation.

- Strongly agree
- Agree
- Neither agree nor disagree
- Disagree
- Strongly disagree

* Organizational actions should improve life for future generations.

- Strongly agree
- Agree
- Neither agree nor disagree
- Disagree
- Strongly disagree

* Everyone in an organization needs to be responsible for accomplishing organizational goals.

- Strongly agree
- Agree
- Neither agree nor disagree
- Disagree
- Strongly disagree

* Leadership processes involve the participation of all organization members.

- Strongly agree
- Agree
- Neither agree nor disagree
- Disagree
- Strongly disagree

* Anticipating the future is one of the most important roles of leadership processes.

- Strongly agree
- Agree
- Neither agree nor disagree
- Disagree
- Strongly disagree

* Good leadership requires that ethical issues have high priority.

- Strongly agree
- Agree
- Neither agree nor disagree
- Disagree
- Strongly disagree

* Successful organizations make continuous learning their highest priority.

- Strongly agree
- Agree
- Neither agree nor disagree
- Disagree
- Strongly disagree

* Environmental preservation should be a core value of every organization.

- Strongly agree
- Agree
- Neither agree nor disagree
- Disagree
- Strongly disagree

* Organizations must be ready to adapt to changes that occur outside the organization.

- Strongly agree
- Agree
- Neither agree nor disagree
- Disagree
- Strongly disagree

* An organization needs flexibility in order to adapt to a rapidly changing world.

- Strongly agree
- Agree
- Neither agree nor disagree
- Disagree
- Strongly disagree

Leadership survey

6. Section 4 (of 4): Leadership Attitudes

* Do you value good leadership?

- A great deal
- A lot
- A moderate amount
- A little
- Not at all

* Do you believe good leadership is important for your managers?

- Strongly agree
- Agree
- Neither agree nor disagree
- Disagree
- Strongly disagree

* Do you believe you are a good leader?

- Very likely
- Likely
- Neither likely nor unlikely
- Unlikely
- Very unlikely

* Do you believe you are an effective leader?

- Very likely
- Likely
- Neither likely nor unlikely
- Unlikely
- Very unlikely

How is this demonstrated?

* Should you aim to improve your leadership knowledge and skills?

- A great deal
- A lot
- A moderate amount
- A little
- None at all

* How much influence does observing others in leadership positions have on your own leadership development?

- A great deal
- A lot
- A moderate amount
- A little
- None at all

* How much influence does practicing particular leadership skills yourself have on your own leadership development?

- A great deal
- A lot
- A moderate amount
- A little
- None at all

* Do you believe a leader must control the group or organization.

- Strongly agree
- Agree
- Neither agree nor disagree
- Disagree
- Strongly disagree

* Do you believe a leader must maintain tight control of the organization.

- Strongly agree
- Agree
- Neither agree nor disagree
- Disagree
- Strongly disagree

* Do you believe a leader should maintain complete authority.

- Strongly agree
- Agree
- Neither agree nor disagree
- Disagree
- Strongly disagree

* Do you believe a leader should take charge of the group.

- Strongly agree
- Agree
- Neither agree nor disagree
- Disagree
- Strongly disagree

* The main tasks of a leader are to make and then communicate decisions.

- Strongly agree
- Agree
- Neither agree nor disagree
- Disagree
- Strongly disagree

* The main task of a leader is to make the important decisions for an organization.

- Strongly agree
- Agree
- Neither agree nor disagree
- Disagree
- Strongly disagree

* Positional leaders deserve credit for the success of an organization.

- Strongly agree
- Agree
- Neither agree nor disagree
- Disagree
- Strongly disagree

* The responsibility for taking risks lies with the leaders of an organization.

- Strongly agree
- Agree
- Neither agree nor disagree
- Disagree
- Strongly disagree

* It is important that a single leader emerge in a group.

- Strongly agree
- Agree
- Neither agree nor disagree
- Disagree
- Strongly disagree

* Members should be completely loyal to the designated leaders of an organization.

- Strongly agree
- Agree
- Neither agree nor disagree
- Disagree
- Strongly disagree

* The most important members of an organization are its leaders.

- Strongly agree
- Agree
- Neither agree nor disagree
- Disagree
- Strongly disagree

* When an organization is in danger of failure, new leaders are needed to fix its problems.

- Strongly agree
- Agree
- Neither agree nor disagree
- Disagree
- Strongly disagree

* Leaders are responsible for the security of organization members.

- Strongly agree
- Agree
- Neither agree nor disagree
- Disagree
- Strongly disagree

* An organization should try to remain as stable as possible.

- Strongly agree
- Agree
- Neither agree nor disagree
- Disagree
- Strongly disagree

7. Any final thoughts/comments on leadership in the biological sciences?

Do you have any additional thoughts/comments about the importance of leadership or leadership training in the biological sciences?
