# Supplementary material for: Profiling leadership: Attitudes, knowledge and training in the biological sciences
Source: PLoS One. 2023 Jun 7;18(6):e0286826. doi: 10.1371/journal.pone.0286826 (PMC10246786; doi:10.1371/journal.pone.0286826)
Supplement: S2 Appendix — (PDF) [file pone.0286826.s016.pdf]

Appendix 2

Previous key work in this area utilised a specific tool, the Leadership Attitudes and Beliefs Scale (LABS) (Wielkiewicz and M., 2000; Fischer et al., 2015). Permission to use the LABS scale was granted by Professor Richard Wielkiewicz (The College of Saint Benedict and Saint John's University, USA) (personal correspondence).

Leadership Perceptions (Systemic Thinking dimension)

These 14 questions quantify “The **Systemic** Thinking” on the LABS scale.

1. An effective organization develops its human resources.
2. Leadership activities should foster discussions about the future.
3. Effective leadership seeks out resources needed to adapt to a changing world.
4. Individuals need to take initiative to help their organization accomplish its goals.
5. Leadership should encourage innovation.
6. Organizational actions should improve life for future generations.
7. Everyone in an organization needs to be responsible for accomplishing organizational goals.
8. Leadership processes involve the participation of all organization members.
9. Anticipating the future is one of the most important roles of leadership processes.
10. Good leadership requires that ethical issues have high priority.
11. Successful organizations make continuous learning their highest priority.
12. Environmental preservation should be a core value of every organization.
13. Organizations must be ready to adapt to changes that occur outside the organization.
14. An organization needs flexibility in order to adapt to a rapidly changing world.

The following 9 questions were added:

- Do you think leadership is important?
- Do you think having formal leadership training/skills is important for lecturers?
- Do you think having formal leadership training/skills is important for research group leaders?
- Should formal leadership training/skills be part of postgraduate education (in the biological sciences)?
- Should gaining formal leadership training/skills be essential for lecturers?
- Should gaining formal leadership training/skills be essential for research group leaders?
- Is having good leadership (in your workplace) important for your personal development?
- Do you believe your line manager/supervisor is a good leader?
- Do you think leadership is something that can be taught?

Leadership Attitudes (Hierarchical Thinking dimension)

These 14 questions quantify "**Hierarchical** Thinking" on the LABS scale.

1. Do you believe a leader must control the group or organization?
2. Do you believe a leader must maintain tight control of the organization?
3. Do you believe a leader should maintain complete authority?
4. Do you believe a leader should take charge of the group?
5. The main tasks of a leader are to make and then communicate decisions.
6. The main task of a leader is to make the important decisions for an organization.
7. Positional leaders deserve credit for the success of an organization.
8. The responsibility for taking risks lies with the leaders of an organization.
9. It is important that a single leader emerge in a group.
10. Members should be completely loyal to the designated leaders of an organization.
11. The most important members of an organization are its leaders.
12. When an organization is in danger of failure, new leaders are needed to fix its problems.
13. Leaders are responsible for the security of organization members.
14. An organization should try to remain as stable as possible.

The following 9 questions were added:

- Do you value good leadership?
- Is good leadership important to you?
- Do you believe good leadership is important for your managers?
- Do you believe you are a good leader?
- Do you believe you are an effective leader?
- ‡ How is this demonstrated? (Free text answer)
- Should you aim to improve your leadership knowledge and skills?
- How much influence does observing others in leadership positions have on your own leadership development?
- How much influence does practicing particular leadership skills yourself have on your own leadership development?
